# Genome‐wide association study of metabolic traits in the giant duckweed *Spirodela polyrhiza*

**DOI:** 10.1111/plb.13747

**Published:** 2024-12-04

**Authors:** M. Höfer, M. Schäfer, Y. Wang, S. Wink, S. Xu

**Affiliations:** ^1^ Institute for Organismic and Molecular Evolution (iomE) Johannes Gutenberg University of Mainz Mainz Germany; ^2^ Institute for Evolution and Biodiversity University of Münster Münster Germany; ^3^ Institute for Quantitative and Computer Biosciences Johannes Gutenberg University of Mainz Mainz Germany

**Keywords:** duckweed metabolism, mGWAS, *Spirodela polyrhiza*, targeted metabolomics, trait variation

## Abstract

- The exceptionally high growth rate and high flavonoid content make the giant duckweed *Spirodela polyrhiza* (L.) Schleid. (Arales: Lemnaceae Martinov) an ideal organism for food production and metabolic engineering. To facilitate this, identification of the genetic basis underlying growth and metabolic traits is essential.
- Here, we analysed growth and content of 42 metabolites in 137 *S. polyrhiza* genotypes and characterized the genetics underpinning these traits using a genome‐wide association (GWA) approach.
- We found that biomass positively correlated with the content of many free amino acids, including L‐glutamine, L‐tryptophan, and L‐serine, but negatively correlated with specialized metabolites, such as flavonoids. GWA analysis showed that several candidate genes involved in processes such as photosynthesis, protein degradation, and organ development were jointly associated with multiple metabolic traits.
- The results suggest the above genes are suitable targets for simultaneous optimization of duckweed growth and metabolite levels. This study provides insights into the metabolic diversity of *S. polyrhiza* and its underlying genetic architecture, paving the way for industrial applications of this plant via targeted breeding or genetic engineering.

## Graphical abstract

A genome‐wide association study in duckweed revealed candidate genes associated with plant growth, photosynthesis, starch metabolism, and protein turnover.

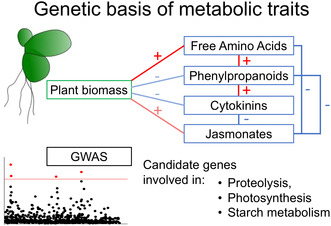

## INTRODUCTION

With a biomass duplication rate of less than three days, *Spirodela polyrhiza* (L.) Schleid. is one of the fastest‐growing angiosperms (Ziegler *et al*., 2015). Due to its high levels of flavonoids and amino acids, *S. polyrhiza* is one of the best organisms for producing food resources and pharmaceuticals (Zhao *et al*., 2015; Acosta *et al*., 2021; Baek *et al*., 2021; Böttner *et al*., 2021; Smith *et al*., 2024). To fully realize its industrial potential, specific improvements in its growth and metabolite production are needed, which require detailed understanding of the genetic mechanisms controlling these traits. Although previous forward genetic studies on metabolic traits in species like *Zea mays* L. (Chen *et al*., 2016), *Oryza sativa* L. (Chen *et al*., 2014; Zhang *et al*., 2019; Cu *et al*., 2021) and *Arabidopsis thaliana* (L.) Heynh. (Angelovici *et al*., 2013; 2017) identified many promising candidate genes, the genetic principles controlling growth and metabolism in *S. polyrhiza* largely remain unknown, hampering its further biotechnological optimization.

**Fig. 1:**
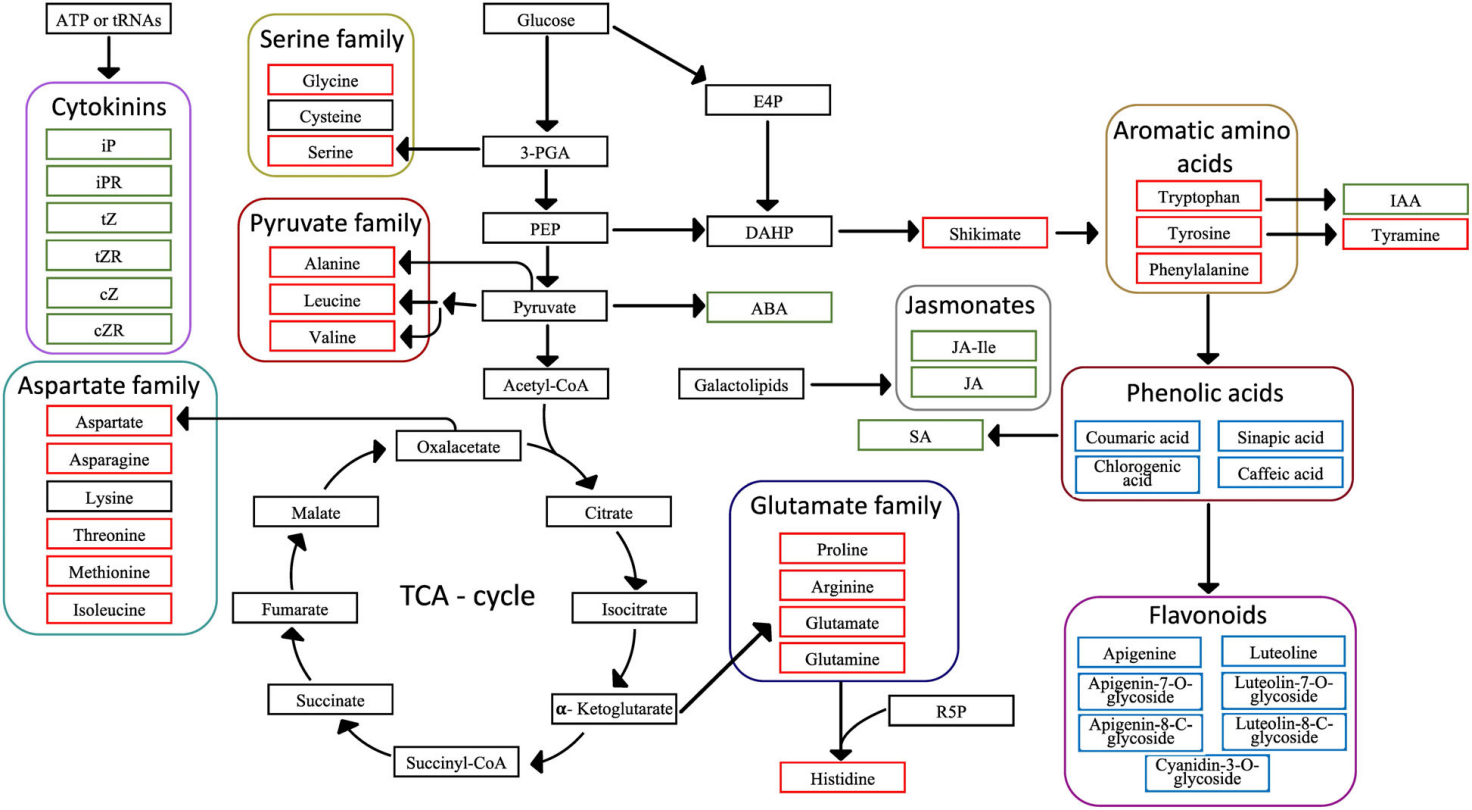
Overview of the biosynthesis of metabolites analysed in this study. Metabolites are connected to their biochemical precursors with arrows. All quantified compounds were sorted into three categories: amino acid metabolism, specialized metabolism, and phytohormones, as shown in boxes coloured red, blue and green, respectively. Direct degradation products and biosynthetic precursors of amino acids such as tyramine and shikimic acid are grouped together with all quantified amino acids in the group amino acid metabolism. Amino acids were subcategorized into five families based on their last common precursor (serine, pyruvate, aspartate and glutamate) or on common chemical properties (aromatic amino acids). All specialized metabolites quantified in this study are phenylpropanoids. Phenolic acids are also precursors of flavonoids. In contrast to amino acids and specialized metabolites, compounds with a major function in signalling were characterized as phytohormones. Chemically derived metabolites with the same precursors were further grouped in biosynthetic families, illustrated by frames of different colours. 3‐PGA = 3‐phosphoglycerate; ABA = abscisic acid; CoA = coenzyme A; cZ = cis‐zeatin; cZR = cis‐zeatin riboside; DAHP = 3‐deoxyarabinoheptulonate‐7‐phosphate; E4P = erythrose‐4‐phosphate; IAA = indole‐3‐acetic acid; iP = isopentenyladenine; iPR = isopentenyladenine riboside; JA = jasmonic acid; JA‐Ile = jasmonic acid‐isoleucine conjugate; PEP = phosphoenolpyruvate; R5P = ribose‐5‐phosphate; SA = salicylic acid; TCA = tricarboxylic acid; tZ = trans‐zeatin; tZR = trans‐zeatin riboside.

For most plants, to survive natural stress factors, such as herbivory, they must carefully allocate resources to growth or defence pathways. On the molecular level, free amino acids play a pivotal role in balancing resource distribution between biomass production and synthesis of defence metabolites. Biosynthesis of amino acids can account for 50% of all carbon compound syntheses and consumes 32% of total fixed carbon dioxide (Smith *et al*., 1961), making them a major carbon sink for biomass production in plants (Noctor & Foyer, 1998). The central metabolic function of free amino acids is highlighted by their association with key metabolic pathways, such as glycolysis, tricarboxylic acid (TCA) cycle, pentose phosphate pathway, urea cycle and photorespiration (Fig. 1) (Noctor & Foyer, 1998). Besides their function in biomass gain, amino acids are precursors of many specialized metabolites like flavonoids and phenolic acids (Fig. 1), which are involved in plant defence. Biosynthesis of specialized metabolites often negatively impacts plant biomass, primarily through consumption of nutrient resources (Züst *et al*., 2011) and autotoxicity (Dick *et al*., 2012; Li *et al*., 2021). Consequently, optimization for high crop yields often leads to high susceptibilities to pests due to a lack of defence metabolites, a phenomenon known as the growth–defence trade‐off (Huot *et al*., 2014; Züst & Agrawal, 2017). On a regulatory level, resource allocation from growth to defence pathways is controlled by phytohormones such as jasmonic acid (JA), abscisic acid (ABA), salicylic acid (SA) and indole‐3‐acetic acid (IAA) (Aftab *et al*., 2010; Huot *et al*., 2014; Živanović *et al*., 2020; Hui *et al*., 2023). Therefore, understanding the genetic mechanisms controlling levels of phytohormones, free amino acids and related metabolites is key for optimizing the biomass yield and specialized metabolite content in plants.

Here, we focus on understanding the genetic control regulating levels of free amino acids and their related specialized metabolites in giant duckweed. We quantified content of 42 metabolites (Fig. 1) in 137 genotypes of *S. polyrhiza* and performed a genome‐wide association study (GWAS) on the content of these metabolites. We specifically aim to address the following questions: (i) to what extent do growth and metabolism vary in *S. polyrhiza*; (ii) which metabolites are associated with the intra‐specific variations of biomass and growth in *S. polyrhiza*; and (iii) which genes control these metabolic traits?

## MATERIAL AND METHODS

### Data collection and sample preparation

In a previous study we estimated the growth of 138 *S. polyrhiza* genotypes under herbicide treatment and under control conditions (Höfer *et al*., 2024b). We quantified growth as relative growth rate of frond area (RGR of FA) and frond number (RGR of FN) and measured biomass as fresh weight (FW) and dry weight (DW) at the end of a 7‐day growth period. For each genotype, growth and biomass were estimated in three technical replicates. The quantified growth and biomass parameters are publicly available (Höfer *et al*., 2024a). A table documenting geographic origin of all genotypes analysed in this study can be found at https://datadryad.org/stash/downloads/file_stream/2894056.

All analyses included in this study are based on plant material grown under control conditions (Höfer *et al*., 2024b). After harvesting, we freeze‐dried plant material and stored it at room temperature (RT) until metabolite extraction. To have sufficient plant material for metabolite analysis, triplicates were pooled for each genotype. The RGRs were estimated (Höfer *et al*., 2024b) based on a published method (Ziegler *et al*., 2015), applying the formula: $\mathrm{RGR}=\frac{\ln\left(N_{t}\right)-\ln\left(N_{0}\right)\;}{t}$, where N_t_ is the frond number or frond area at the endpoint of the assay, *N*_*0*_ is the frond number or frond area at the start of cultivation and t is the duration of the bioassay.

### Metabolite extraction

To investigate the genotype variation in metabolite content via liquid chromatography ‐ mass spectrometry (LC‐MS), we extracted 42 metabolites from pooled triplicates of 137 genotypes (Table S1, Fig. 1). The number of analysed genotypes was reduced from 138 to 137 due to mislabelling of one genotype, which was excluded from all analyses. The protocols used for LC–MS analysis of metabolite levels are described in Table S1–S5. The entire extraction and quantification procedure is based on a previously published method (Schäfer *et al*., 2016).

The samples were ground with two steel balls at 30 Hz for 30 s in a TissueLyser (Qiagen, Venlo, Netherlands). Next, 10 mg aliquots of the homogenized samples were placed in 96‐well BioTubes (Axygen, New York, NY, USA) and 800 μL extraction buffer (methanol:water:formic acid, 15:4:1 (v/v/v)) containing [^13^C_6_]‐IAA (1 ng), [^2^H_5_]‐JA (10 ng), [^2^H_6_]‐ABA (10 ng), [^2^H_4_]‐SA (10 ng), [^2^H_5_]‐tZ (0.5 ng), [^2^H_6_]‐iP (0.2 ng) and [^2^H_6_]‐iPR (0.2 ng) were added to each tube. Tubes were sealed with a pre‐cooled mat and homogenized in a TissueLyser at 25 Hz for 60 s. Samples were incubated at −20 °C overnight then the homogenization step was repeated at 25 Hz for 60 s. We then centrifuged the samples at 2,250 *g* for 20 min at 4 °C and transferred 600 μL of the supernatant in new BioTubes. The pellet was kept for re‐extraction. We diluted an aliquot of 1 μL supernatant in 99 μL algae amino acid standard mix −^13^C, ^15^N (0.5 ng·μL^−1^) (Sigma‐Aldrich, St. Louis, MO, USA) and transferred the dilutions into 96‐well PCR plates (Kisker, Steinfurt, Germany). The PCR plates were then stored at −20 °C till analysis of all amino acids and several secondary metabolites (Methods S1 and S2, Table S1). The pellet was reextracted with 600 μL extraction buffer without standards, homogenized at 25 Hz for 60 s before incubation at −20 °C for 30 min. After another centrifugation step at 2,250 *g* for 20 min at 4 °C, we transfered 600 μL of supernatant from the reextracted samples to the remaining supernatant from the first part of the extraction. We centrifuged the combined supernatants at 2,250 *g* for 20 min at 4 °C. Before loading samples, we conditioned the HR‐X columns (Macherey‐Nagel, Düren, Germany) with 600 μL MeOH, followed by 600 μL extraction buffer and discarded the flow through. We collected the flow‐through of our samples in Nunc 96‐well Deep Well Plates (Thermo Fisher Scientific, Waltham, MA, USA). Next, we added another 200 μL extraction buffer and collected the flow through in the same well plate. We then evaporated the MeOH at 45 °C. Next, we added 850 μL of 1 N formic acid to each sample, mixed the samples at 20 Hz for 30 s using a TissueLyser and centrifuged them at 2,250 *g* for 20 min at 4 °C.

Before starting the next purification step, we first conditioned HR‐XC columns with 600 μL MeOH and then with 600 μL 1 N formic acid and discarded the flow‐through. We loaded samples on the conditioned HR‐XC columns and subsequently washed the columns with 1.2 mL 1 N formic acid, discarded the flow through and eluted the sample with 1 mL 0.2 N formic acid in 80% (v/v) MeOH. We collected the eluates in BioTubes. After sample homogenization by inversion, we transferred 50 μL supernatant into a 96‐well PCR plate for analysis of phytohormones (Method S3, Table S1). The sealed plates were stored at −20 °C until analysis.

For analysis of IAA and phenolic acids (Method S4), the remaining eluate was evaporated under a constant nitrogen stream at 45 °C and samples were reconstituted to 50 μL with 0.2 N HCOOH in 80% (v/v) MeOH. We covered the plates before mixing at 20 Hz for 30 s. After centrifugation at 2,250 *g* for 20 min, samples were transferred to a 96‐well PCR plate and stored at −20 °C till analysis. For extraction of cytokinins (Method S5), we washed the HR‐X column twice with 1 mL 0.35 N NH_4_OH and discarded the flow through. Cytokinins were finally eluted with 1 mL 0.35 N NH_4_OH in 60% (v/v) methanol. Samples were then dried under constant nitrogen flow before being reconstituted to 50 μL with 0.1% (v/v) acetic acid. The samples were then homogenized at 20 Hz for 30 s, before incubation in an ultrasonic bath for 5 min. Samples were then centrifuged at 2,250 *g* for 20 min at 4 °C, the supernatant was transferred to a 96‐well PCR plate and stored at −20 °C until analysis (Method S5).

### LC–MS analysis of metabolite contents

We quantified 42 metabolites via LC–MS. For separation and quantification, we used a Nexera X3 UHPLC system (Shimadzu, Kyoto, Japan) coupled to an LCMS‐8060 system (Shimadzu, Kyoto, Japan). For separation of all metabolites, we used a Zorbax RRHD Eclipse XDB‐C_18_ column (50 × 3 mm, 1.8 μm) (Agilent, Santa Clara, CA, USA) with a 1290 Infinity II inline filter (0.3 μm) (Agilent, Santa Clara, CA, USA). For all measurements, the autosampler was pre‐cooled to 5 °C during the entire measurement procedure. As mobile phase, we used 0.05% (v/v) formic acid and 0.1% (v/v) acetonitrile as solvent A and 100% methanol as solvent B, that were applied in gradient mode at a constant flow rate of 0.5 ml·min^−1^ in all methods. The column oven temperature was always 42 °C. For analyses, we used an electrospray ionization source with the following parameters: nebulizing gas flow: 3 l·min^−1^, heating gas flow: 10 l·min^−1^, drying gas flow: 10 l·min^−1^, interface temperature: 300 °C, DL temperature: 250 °C, heat block temperature: 400 °C and CID gas flow: 270 kPa. For metabolites measured in positive and negative ionization modes, we applied interface voltages of 4000 V and −3000V, respectively (Table S2–S6). A detailed description of individual metabolite analysis can be found in Methods S1–S5.

### Correlation and principal components analysis (PCA)

Next, we used regression and PCA to identify correlation patterns between metabolic parameters and to identify influences of population structure on metabolite levels and growth. We established multiple correlations for all metabolite and growth data using the ggcorrplot R‐package (v 0.1.4.1) (Kassambara, 2023). We used Pearson correlation coefficient to measure strength of linear correlations. All PCAs were conducted with the R package pcaMethods (v 1.88.0) (Stacklies *et al*., 2007). We calculated 95% confidence intervals based on standard deviations using the vegan package (v 2.6‐2) (Oksanen *et al*., 2022) for all PCAs. All three‐dimensional scatter plots were created using the R package scatterplot3d (v 0.3.42) (Ligges & Mächler, 2003). To identify metabolite categories, we sorted all 42 metabolites into nine groups based on their chemical characteristics and biochemical precursors: flavonoids, phenolic acids, aromatic amino acids, glutamate family, jasmonates, serine family, pyruvate family, aspartate family, and cytokinins (Fig. 1). A PCA on metabolite concentrations was subsequently done for all 137 genotypes.

Since our lab has identified four genetic populations for *S. polyrhiza* (Xu *et al*., 2019; Wang *et al*., 2024), we next investigated to what extent differences in metabolite concentrations and growth are explained by population structures. Assignment of genotypes to different genetic populations was done previously. Due to the high proportion of clonal genotypes in our accession, 137 genotypes were grouped into 97 clonal families for PCA on population structure of growth parameters and metabolite levels, respectively. For analysis, each clonal family was represented by the genotype with the highest sequencing coverage, as described previously (Höfer *et al*., 2024b).

### Genome‐wide association study

To explore the genetic basis underlying metabolic traits, we conducted GWAS on growth data and free metabolite content of all representative genotypes. We used single nucleotide polymorphisms (SNPs) and structural variations (SVs) (>50 bp) as genetic markers for all GWAS analyses. To allow their use for GWAS, SVs were re‐coded according to a previously published method (Lemay & Malle, 2022). To correct for missing data, we performed an imputation of both SNP and SV datasets using beagle 5.4 v 22Jul22.46e (Browning *et al*., 2018).

We conducted GWAS on SNP and SV data using the vcf2gwas platform (v 0.8.7) (Vogt *et al*., 2021). Markers were pruned using the Plink software integrated into vcf2gwas, with phased *r* ^2^ thresholds of 0.33 and 0.15 for SVs and SNPs, respectively. For filtering low abundance alleles, we applied a minor allele frequency (MAF) threshold of 5%, leaving 10,057 SNPs and 1,182 SVs for analysis. We corrected for population structure through PCA from the input genotype files. Here, we estimated population structure based on four PCs accounting for the four genetic populations of *S. polyrhiza*. All GWAS were conducted using a univariate linear mixed model (Zhou & Stephens, 2012). The genotype data, including annotation, were published previously (Wang *et al*., 2024) and can be found at: https://github.com/Xu‐lab‐Evolution/Great_duckweed_popg (accessed 22 March 2024).

### RT‐qPCR quantification of candidate genes

Our GWAS detected a deletion in *SpUBP7* that was associated with increased content of L‐glutamine and L‐serine. To validate the effect of the deletion on gene function, we studied expression of *SpUBP7* (*SpGA2022_056000*) in two different allelic backgrounds.

For this, we cultivated genotypes SP012 (low content of L‐serine and L‐glutamine, no deletion in *SpUBP7*) and SP187 (high content of L‐serine and L‐glutamine, homozygous for deletion in *SpUBP7*) in N‐medium (Appenroth, 2015) at 26 °C, 135 μmol photons m^−2^·s^−1^ 16/8 h light/dark photoperiod. For each genotype, five replicates were made, each consisting of ten fronds growing as colonies. The fronds were cultivated in plastic beakers (product 560/250, transparent, round, 250 mL, Plastikbecher.de, Giengen, Germany), covered with perforated lids to allow gas exchange. Each beaker was filled with 150 mL N‐medium.

After five days of cultivation, we separately harvested root and frond tissue of each replicate for RNA extraction. We extracted RNA from <20 mg fresh weight using the InnuPREP RNA mini kit (Analytik, Jena, Germany). For each sample, RNA concentration was measured using a Nanodrop and integrity was checked via gel electrophoresis on a 1% agarose gel. We used 600 ng RNA for each cDNA synthesis following instructions of the RevertAid First Strand cDNA synthesis kit (Takara, Shiga, Japan). For all cDNA syntheses, we used oligo‐dT primers. qPCRs were carried out using a RotorGene Q system (Qiagen, Venlo, Netherlands) applying a time program with an initial denaturation of 98 °C for 3 min, followed by 40 cycles with 98 °C for 3 s and 60 °C for 20 s. Before qPCR, we estimated primer efficiency for *SpUBP7* using serial dilutions of pooled cDNA templates from root and frond tissue samples. Primer specificity was checked by evaluating qPCR products on a 2% agarose gel and with melting curve analysis. We conducted all qPCR reactions according to instructions of the KAPPA SYBR FAST kit (Roche, Basel, Switzerland). All cDNA samples were diluted at 1:100 in nuclease‐free water before analysis. We used *ALPHA‐ELONGATION FACTOR 1 SpaEF* (*SpGA2022_005771*) and *GLYCERINALDEHYDE‐3‐PHOSPHATE DEHYDROGENASE SpGAPDH* (*SpGA2022_054082*) as references for visualization of candidate gene expression according to the delta–delta Ct method with multiple reference genes, as published previously (Hellemans *et al*., 2007). The primers used can be found in Table S7. Primers for the reference genes *SpaEF* and *SpGAPDH* were used in previous publications (Wang *et al*., 2024; Höfer *et al*., 2024b).

### Software and statistics

We conducted all statistical analyses with R v 4.2.0. Standard errors of means were calculated using the plotrix R package (Lemon, 2006). For all LC–MS measurements, we recorded and quantified analyte and standard peaks with LabSolutions software v 5.97. We estimated the broad sense heritability (H2.c) from our comprehensive dataset, which includes repeatedly analysed genotypes with the lme4 R package (Bates *et al*., 2015) following a previously published method (Cullis *et al*., 2006).

The significance of correlations was evaluated using the *F*‐test. We used Bonferroni corrected *P* < 0.05 to determine significant genetic markers associated with our traits. The *P*‐values for GWAS were calculated using the Wald test. For *SpUBP7*, we compared the SV effect of heterozygous and WT samples using a two‐tailed Student's *t*‐test. We used Student's *t*‐test for comparison of gene expression between two genotypes. We conducted the Levene test to check for homogeneity of variances using the car R package (Fox & Weisberg, 2019).

## RESULTS

### Intraspecific variation of growth rate and metabolic traits

We quantified 42 metabolites, including 20 free amino acids and their derivatives, 11 specialized metabolites, and 11 phytohormones (Fig. 1) among 137 *S. polyrhiza* genotypes. L‐Asparagine and L‐glutamine were the most abundant free amino acid in *S. polyrhiza*, with average genotype tissue concentrations of 114.4 and 57.0 μmol·mg^−1^ DW, respectively (Table S8). ABA was the least abundant metabolite, with an average concentration of 37.0 pmol·mg^−1^ DW (Table S9).

Among all quantified metabolic features, the content of flavonoids and phenolic acids had highest intraspecific variation (Table S10, Figures [Link], [Link], [Link], [Link]). Chlorogenic acid and luteolin‐7‐O‐glycoside showed 214.9‐ and 183.8‐fold differences, respectively, among genotypes (Table S10, Figure S3). Growth rate, quantified either as RGR of FN or FA, had <2.4‐fold differences (Figure S1).

On average, growth and biomass had higher broad‐sense heritability than metabolite levels (Table 1, Tables S8–S10). All growth and biomass parameters had overall high broad‐sense heritability values between 0.6 and 0.9, with fresh weight being most heritable (Table 1). A total of 25 metabolites had high heritability values (H2.c > 0.3), while the remaining 16 metabolites had low heritability (H2.c < 0.3) (Tables S8–S10). Together, these findings suggest that growth rate and metabolic traits vary within *S. polyrhiza*, and most of these traits are heritable.

**Table 1:** Phenotype variation and heritability of biomass and growth rate in *S. polyrhiza*.

Fitness trait	Mean	SE	Fold change	H2.c
Fresh weight	227.7 mg	7.3 mg	10.4	0.865
Dry weight	16.67 mg	0.5 mg	10.4	0.602
RGR of FA	0.323 d^−1^	0.004 d^−1^	2.4	0.841
RGR of FN	0.259 d^−1^	0.003 d^−1^	2.2	0.802

H2.c = broad sense heritability according to Cullis *et al*. (2006); RGR of FA = relative growth rate of frond area; RGR of FN = relative growth rate of frond number; SE = standard error.

### Metabolic traits correlate with biomass and growth

**Fig. 2:**
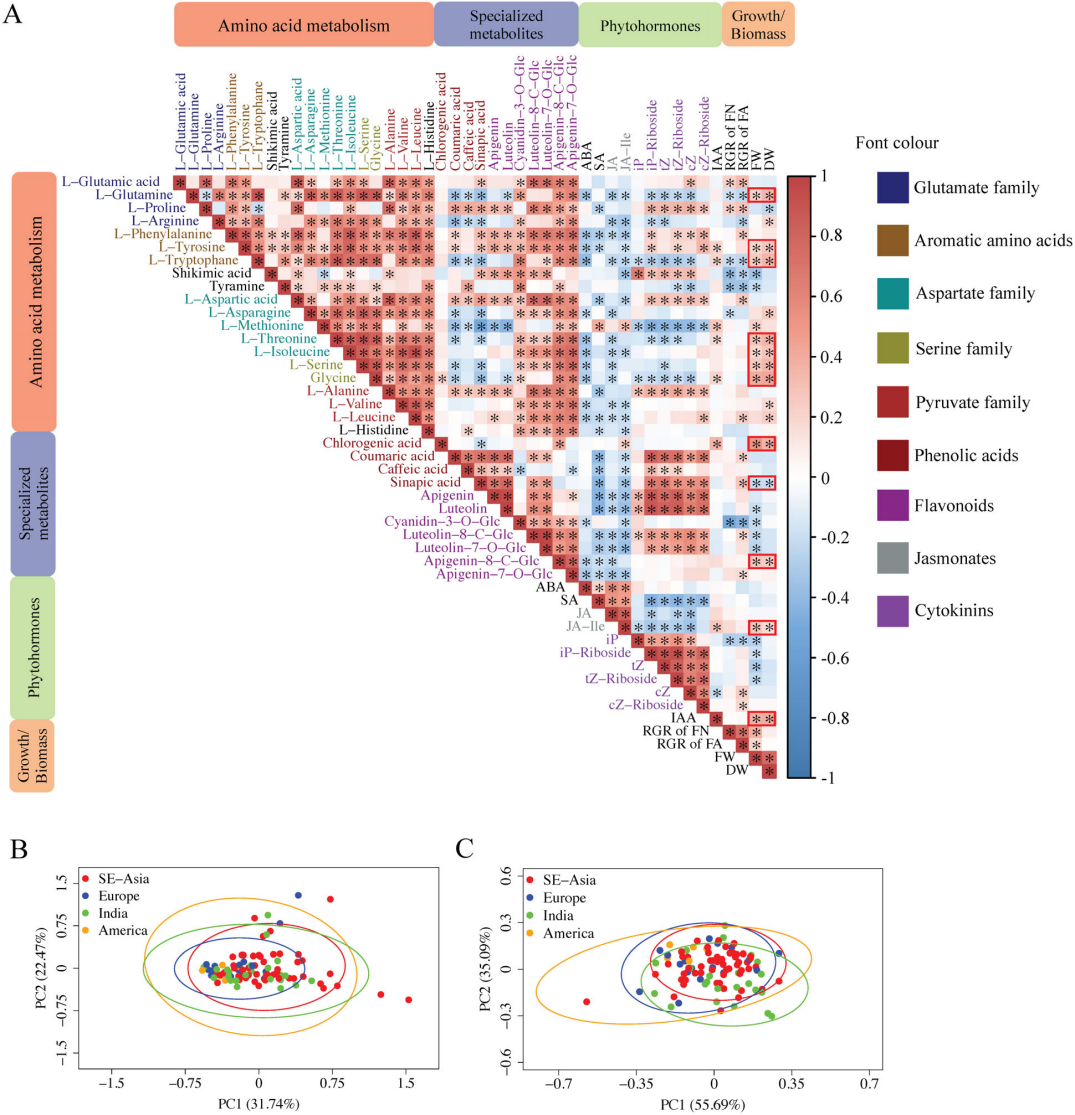
Correlation matrix (A) and principal coordinates analyses (PCA) on growth parameters (B) and metabolite content (C). (A) Tissue concentrations of free amino acids showed strong positive correlation patterns. In most cases, content of free amino acids was positively correlated with flavonoid content and biomass. Metabolite concentrations and growth rates were measured for 137 genotypes. All metabolites were categorized according to Fig. 1. The strength of the correlation was estimated using Pearson correlation coefficient and is reflected by the intensity of red colour for positive and blue for negative correlations. Asterisks highlight significant correlations (*F*‐test, *P* < 0.05). Simultaneous correlations of dry and fresh weight are highlighted with red margins. (B and C) Population‐based differences in growth (B) and metabolite levels (C). All PCAs were conducted on 97 representative genotypes assigned to four previously defined genetic populations (Xu *et al*., 2019). The ellipses show 95% confidence intervals calculated based on standard deviation. The colours of ellipses correspond to the genetic populations. ABA = abscisic acid; cZ = cis‐zeatin; cZR = cis‐zeatin riboside; DW = dry weight; FW = fresh weight; IAA = indole‐3‐acetic acid; iP = isopentenyladenine; iPR = isopentenyladenine riboside; JA = jasmonic acid; JA‐Ile = jasmonic acid‐isoleucine conjugate; RGR of FA = relative growth rate of frond area; RGR of FN = relative growth rate of frond number; SA = salicylic acid; tZ = trans‐zeatin; tZR = trans‐zeatin riboside.

Plant metabolic traits are often associated with growth and biomass (Angelovici *et al*., 2013; Chen *et al*., 2016; Živanović *et al*., 2020; Cu *et al*., 2021). In *S. polyrhiza*, metabolites showed stronger correlations with biomass than growth rate. In total, 19% and 29% of quantified metabolites correlated significantly with growth rate (RGRs of FN and FA) and biomass (FW and DW) (Fig. 2A, Figures S5 and S6), respectively. Among 20 quantified free amino acids, 11 had a significant positive correlation with biomass (DW), including L‐glutamine, L‐tyrosine, L‐serine and L‐threonine (Fig. 2A, Figure S6), whereas five were positively correlated with growth rate (RGR of FN). For phytohormones, only IAA and JA‐Ile were positively correlated with biomass. Interestingly, most of the quantified specialized metabolites, while positively correlated with cytokinins, were negatively correlated with biomass (Fig. 2A). Among all metabolites, cyanidine‐3‐C‐glycoside had the strongest negative correlation with both growth rate and biomass (FW) (Fig. 2A, Figures S5 and S7).

For all quantified metabolites, there was a strong correlation within each metabolite group (Fig. S8). Most free amino acids were positively correlated with each other and with flavonoids. Often, phenolic acids were positively correlated with flavonoids (Fig. 2A), reflecting their biosynthetic relationships (Fig. 1).

Because *S. polyrhiza* has a strong population structure (Xu *et al*., 2019; Wang *et al*., 2024), which can confound phenotypic variations and reduce the power of GWAS, we further investigated whether metabolism differed among populations, using a PCA but no population‐specific differences were found at metabolic level (Fig. 2B, C), indicating GWAS can be used to study most metabolic traits in *S. polyrhiza*.

### Genome‐wide association studies on metabolite traits

We used a GWA approach to identify the genetic basis underlying different metabolic traits. Here, we focused on 10,057 unlinked SNPs and 1,182 SVs, of which 75 SNPs and 15 SVs were significantly associated with the 42 metabolites (Tables S11–S13). Among them, 13 SNPs and three SVs were associated with several metabolites, indicating their pleiotropic role in metabolism. Surprisingly, we did not find any SNPs and only one SV, located in an intergenic region (>30 Kb from next open‐reading frame), were associated with growth rate and biomass (Tables S12 and S13).

**Fig. 3:**
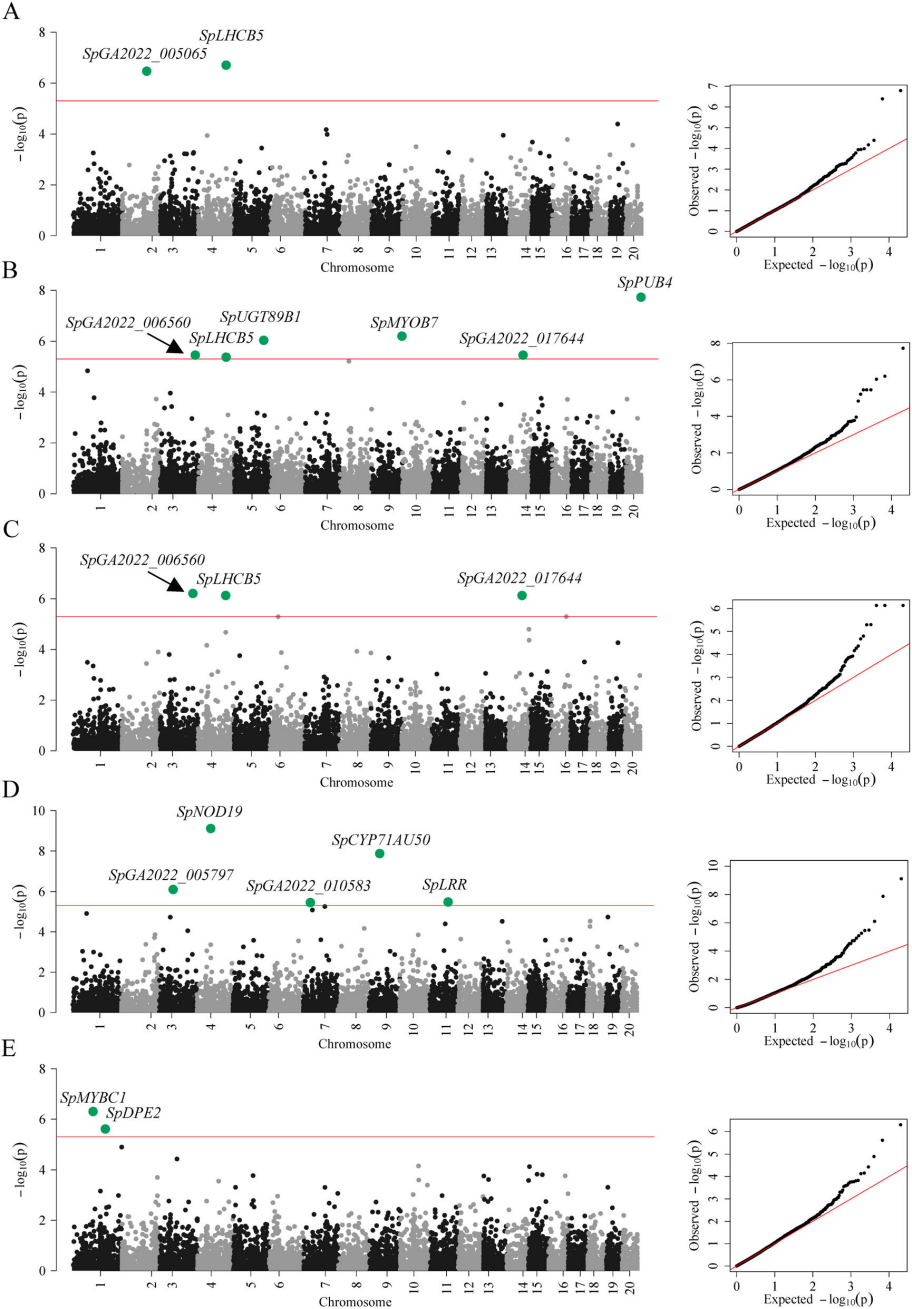
Manhattan plots of SNP‐based GWAS for free amino acids. Each plot shows the GWAS result on concentrations of L‐glutamine (A), L‐tyrosine (B), L‐tryptophan (C), chlorogenic acid (D) and indole‐3‐acetic acid (IAA) (E) with their corresponding QQ plots on the right side. Bonferroni corrected *P* = 0.05 (Wald‐test) at 4.97 10^−6^ was used as significance threshold and is shown as a red line. All significant markers are labelled with the names of gene candidates.

**Fig. 4:**
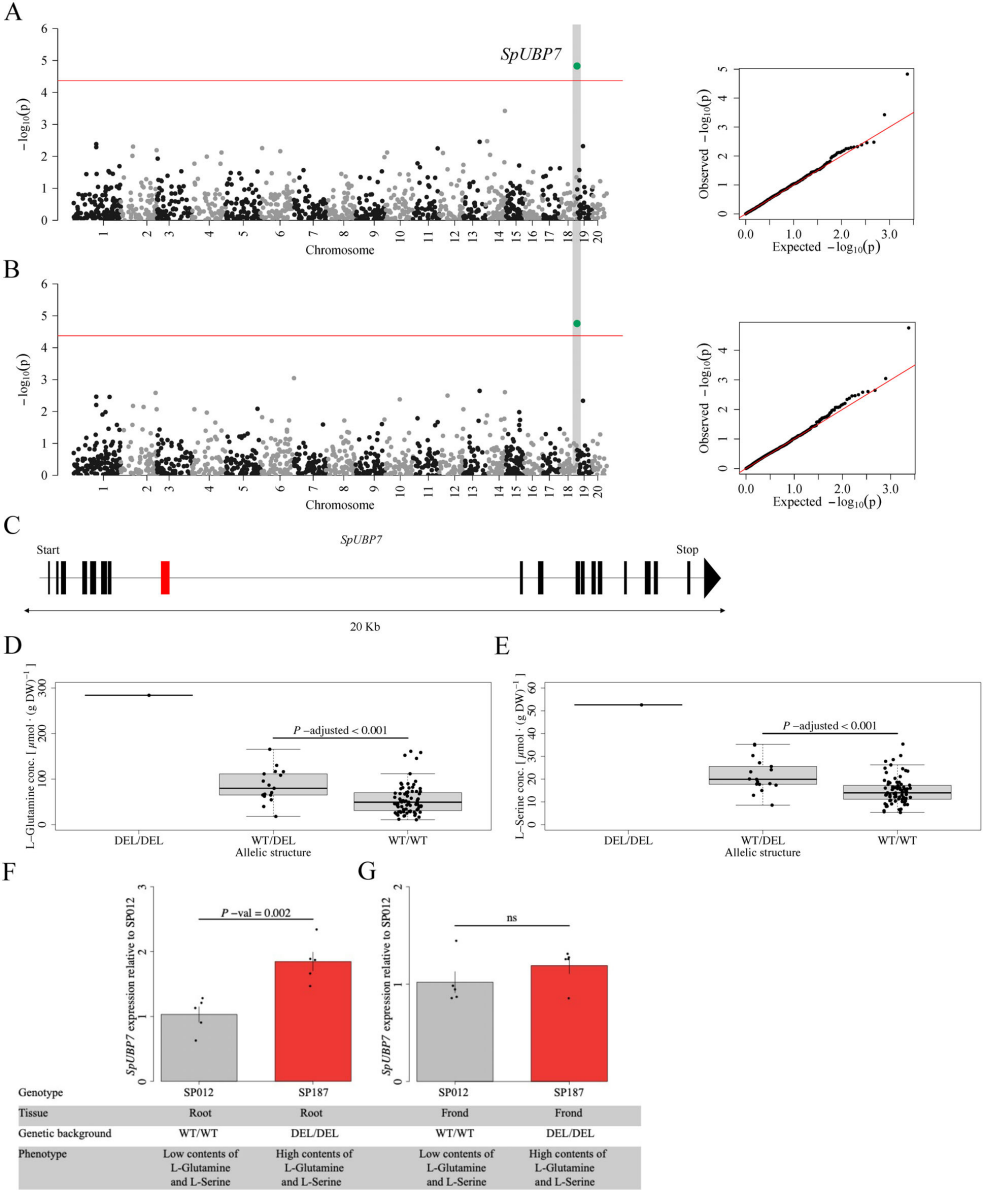
The presence of the intronic deletion in *SpUBP7* is associated with increased content of L‐glutamine and L‐serine. SV‐based GWAS on L‐glutamine (A) and L‐serine (B). The red line represents Bonferroni corrected *P* = 0.05 (Wald‐test) at 4.23 10^−5^. (C) The associated 94 bp deletion (red bar) is located within the 7^th^ intron of *SpUBP7*. Presence of the deletion is associated with increased L‐glutamine (D) and L‐serine (E) content. (F) *SpUBP7* shows genotype‐dependent expression differences at root level. SP187, a genotype that is homozygous for the deletion, shows enhanced *SpUBP7* expression compared to genotype SP012 that lacks the deletion. (G) No significant differential expression of *SpUBP7* between SP187 and SP012 was found in fronds.

Among metabolic traits, several loci were associated with content of L‐glutamine, L‐tyrosine, L‐tryptophan, L‐serine, chlorogenic acid and IAA. Most notably, several SNPs located within the *LIGHT HARVESTING COMPLEX OF PHOTOSYSTEM II 5* (*SpLHCB5*), a structural component of photosystem II (de Bianchi *et al*., 2008), were associated with content of L‐glutamine, L‐valine, L‐tryptophan and L‐tyrosine (Fig. 3A–C, Table S13). Further, an SNP upstream of a *PLANT U‐BOX CONTAINING PROTEIN 4* (*SpPUB4*), which functions as a regulator of cell division in meristematic regions (Kinoshita *et al*., 2015), was associated with L‐tyrosine content. Three SNPs associated with the content of L‐tyrosine, chlorogenic acid and IAA were linked to *SpUGT89B1*, *SpCYP71AU50* and *SpMYBC1*, three genes functioning in secondary metabolite biosynthesis (Caputi *et al*., 2012; Yamaguchi *et al*., 2014; Ke *et al*., 2021) (Fig. 3B,D,E). An SNP within *SpDPE2*, which is functionally involved in starch metabolism, is associated with IAA content (Li *et al*., 2022) (Fig. 3E). For SVs, the content of L‐glutamine and L‐serine were associated with a 94‐bp intronic deletion in *UBIQUITIN‐SPECIFIC PROTEASE 7 SpUBP7* (Fig. 4A–C). Homologues of *SpUBP7* have been shown to stabilize ubiquitin upon proteasome binding and regulate proteasomal activity (Leggett *et al*., 2002; Wu *et al*., 2019). Presence of the intronic deletion was associated with increased content of L‐glutamine and L‐serine (Fig. 4D,E) and increased expression of *SpUBP7* in roots (Fig. 4F,G). These findings suggest that biomass and metabolite content are coordinated through photosynthesis, starch metabolism, cell division, secondary metabolite biosynthesis, and protein degradation.

## DISCUSSION

Using targeted metabolomics on 137 *S. polyrhiza* genotypes, we identified strong positive correlations between chemically related metabolites. Additionally, candidate genes involved in photosynthesis (*SpLHCB5*), protein degradation (*SpUBP7*) and organ development (*SpLOB*, *SpAGL62*) were repeatedly associated with different metabolite levels, suggesting strong genetic co‐regulation. Since levels of free amino acids and specialized metabolites correlated with plant biomass in many cases, some of these candidate genes are potentially of use for joint optimization of biomass and nutrient level.

Free amino acids have large pool sizes and turnover rates in duckweed and serve as major carbon and nitrogen sources for starch and storage protein production (Evans *et al*., 2018). Previous work found that supplementation of growth medium with amino acids such as L‐glutamine was associated with increased protein content and biomass in duckweed (Shi *et al*., 2023). In agreement with these observations, associations between free amino acid levels and biomass were mostly positive. Compared to free amino acids, phenylpropanoids and phytohormones have higher functional diversity. In *S. polyrhiza*, anthocyanins alleviate Cr(VI) stress (Oláh *et al*., 2009), whereas luteolins and apigenins are involved in stress responses to copper and UV‐light, respectively (Böttner *et al*., 2021). The content of these three flavonoid classes is inversely correlated with biomass in *S. polyrhiza* (Oláh *et al*., 2009; Böttner *et al*., 2021), which can be explained by increased allocation of carbon resources to flavonoid biosynthesis. In comparison, our study revealed more diverse association patterns of flavonoid levels with biomass. While the content of sinapic acid, luteolins and cyanidin‐3O‐glycoside was negatively associated with biomass, content of apigenin‐8‐C‐glycoside and chlorogenic acid showed positive correlations with biomass. In other plant species, changes in profiles of glycosylated flavonoids regulate plant growth by influencing auxin transport (Ringli *et al*., 2008), possibly explaining different association patterns of glycosylated luteolins and apigenins to plant biomass in our study. Content of chlorogenic acid was associated with increased leaf growth and content of antioxidants and proteins, suggesting a regulatory function in plant growth (Zhang *et al*., 2024). The overall positive association of cytokinin levels with most phenylpropanoids was previously explained by their function in coordinating resource allocation to defence metabolism during plant development (Brütting *et al*., 2017). For phytohormones, levels of JA‐Ile were positively correlated with biomass. In duckweeds, jasmonates show a bimodal dose–response pattern, with small concentrations stimulating growth and turion germination, and high concentrations delaying growth (Appenroth *et al*., 1991; Piotrowska *et al*., 2010). Since metabolites were measured in unstressed plant material, JA content likely never exceeded constitutive levels, which would be required for significant growth inhibition. Together, these correlation patterns suggest that *S. polyrhiza* can be simultaneously optimized for higher biomass and increased content of certain phenylpropanoids.

On a genetic level, we identified for most specialized metabolite and phythohormone levels genetic associations, whereas for many free amino acids, no significant associations were found. For growth rate and biomass, we did not find significant associations for any markers located in genic regions, likely due to their highly polygenic features. Previous GWAS on complex traits found that success in identifying genetic associations greatly depends on sample size and phenotype variations (Chen *et al*., 2014; Schizophrenia Working Group of the Psychatric Genomics Consortium, 2014; Duncan *et al*., 2019). In line with previous reports (Chen *et al*., 2016; Pham *et al*., 2023), secondary metabolite levels had the highest average phenotypic variation across all metabolic traits, whereas variation in growth parameters was lowest. For specialized metabolites, high variation in tissue concentration was explained by low trait complexity (Chen *et al*., 2014; Haghi *et al*., 2022; Pham *et al*., 2023). While primary metabolites and growth are regulated by many small effect loci, specialized metabolites are often controlled by a few major‐effect loci (Chen *et al*., 2014, 2016). Since small effect loci are often diffusely distributed across the genome, GWAS on primary metabolite levels and growth often require large sample sizes to detect them (Duncan *et al*., 2019). Compared to other association studies on plant metabolic traits (Angelovici *et al*., 2013; Chen *et al*., 2014, 2016; Cu *et al*., 2021), our GWAS suffered from a low sample size, likely explaining the lack of genetic associations for growth traits and several metabolites.

On a physiological level, differences in growth were correlated with photosynthesis rate in duckweed (Sree *et al*., 2015). Hence, metabolism of storage molecules plays a major role in plant growth. Interestingly, many biomass‐correlated metabolites were associated with genes influencing photosynthetic efficiency (*SpLHCB5*), protein turnover (*SpUBP7*), starch metabolism (*SpDPE2*) and cell‐cycle control (*SpPUB4*). These genes provide suitable candidates for future reverse genetic studies, which are required for further elucidation of their exact function in duckweed metabolism. Taken together, this study provides insights into the molecular basis of growth regulation and metabolite homeostasis in *S. polyrhiza*. Through identification of gene candidates associated with metabolic traits, this study contributes to laying the foundations for further optimization strategies in *S. polyrhiza*.

## AUTHOR CONTRIBUTIONS

MH analysed the data. MH, MS, SW, and YW performed the experiments. YW and SX provided resources and technical infrastructure. SX and MH conceived and supervised the project. MH wrote the manuscript. All authors contributed to the final version of the manuscript.

## CONFLICT OF INTEREST

The authors declare that the research was conducted in the absence of any commercial or financial relationships that could be construed as a potential conflict of interest.

## FUNDING INFORMATION

This research was funded by the “Deutsche Forschungsgemeinschaft”, grant number 427577435 and 435681637 to SX.

## Supporting information


**Figure S1.** Kernel density distribution of growth and biomass parameters measured for 137 genotypes of *S. polyrhiza*: Fresh weight (A), RGR of frond number (B), RGR of frond area (C) and dry weight (D) for 137 genotypes of *S. polyrhiza*.

**Figure S2.** Kernel density distribution of free amino acid concentrations measured for 137 genotypes of *S. polyrhiza*: L‐Alanine (A), L‐Arginine (B), L‐Asparagine (C), L‐Aspartic acid (D), L‐Glutamic acid (E), L‐Glutamine (F), L‐Isoleucine (G), L‐Leucine (H), L‐Phenylalanine (I), L‐Proline (J), L‐Serine (K), L‐Threonine (L), L‐Tryptophan (M), L‐Valine (N), Glycine (O), L‐Histidine (P), L‐Methionine (Q), L‐Tyrosine (R), Shikimic acid (S) and Tyramine (T).

**Figure S3.** Kernel density distribution of secondary metabolite concentrations measured for 137 genotypes of *S. polyrhiza*: Chlorogenic acid (A), Coumaric acid (B), Caffeic acid (C), Sinapic acid (D), Apigenin (E), Luteolin (F), Cyanidin‐3‐O‐glycoside (G), Luteolin‐7‐O‐glycoside (H), Luteolin‐8‐C‐glycoside (I), Apigenin‐8‐C‐glycoside (J) and Apigenin‐7‐O‐glycoside (K).

**Figure S4.** Kernel density distribution of phytohormone concentrations measured for 137 genotypes of *S. polyrhiza*: Abscisic acid (A), Salicylic acid (B), Jasmonic acid (C), Jasmonic acid‐Isoleucine conjugate (D), N^6^–Isopentenyladenine (E), N^6^–Isopentenyladenine riboside (F), trans‐Zeatin (G), trans‐Zeatin riboside (H), cis‐Zeatin (I), cis‐Zeatin riboside (J), Indole‐3‐acetic acid (K).

**Figure S5.** Scatterplots of all significant correlations (*F*‐test, *P* ≤ 0.05) of fresh weight with individual free metabolite contents. Fresh weight significantly correlated with contents of L‐Glutamine (A), L‐Tyrosine (B), L‐Tryptophane (C), L‐Threonine (D), L‐Isoleucine (E), L‐Serine (F), Glycine (G), Chlorogenic acid (H), Sinapic acid (I), Luteolin (J), Cyanidin‐3‐O‐glycoside (K), Luteolin‐8‐C‐glycoside (L), Luteolin‐7‐O‐glycoside (M), Apigenin‐8‐C‐glycoside (N), Jasmonic acid–Isoleucine (O) and Indole‐3‐acetic acid (P).

**Figure S6.** Scatterplots showing all identified significant correlations (*F*‐test, P ≤ 0.05) of individual free metabolite contents with dry weight. Dry weight was significantly correlated with levels of L‐Glutamine (A), L‐Tyrosine (B), L‐Tryptophane (C), L‐Threonine (D), L‐Isoleucine (E), L‐Serine (F), Glycine (G), Chlorogenic acid (H), Sinapic acid (I), Apigenin‐8‐C‐glycoside (J), Jasmonic acid–Isoleucine (K), Indole‐3‐acetic acid (L).

**Figure S7.** Correlation of growth with contents of Cyanidin‐3‐O‐glycoside. Both metabolites showed strong negative correlation patterns with RGR of frond number (Pearson, ρ = −0.57) (A) and RGR of frond area (Pearson, ρ = −0.52) (B).

**Figure S8.** Principal coordinate analysis (PCA) on levels of 42 metabolites, that were categorized into nine groups according to their biosynthetic origin. Contents of all free metabolites except that of salicylic acid (SA) and Jasmonates (JA and JA‐Ile) formed a cluster.

**Data S1.** Supplemental Methods.

**Data S2.** Supplementary Tables.

## Data Availability

All raw data and scripts used for analysis can be found at: https://doi.org/10.17632/xwsxpfcysd.1
