# Supplementary figures and images for: Genome‐wide association study of metabolic traits in the giant duckweed Spirodela polyrhiza
Source: Plant Biol (Stuttg). 2024 Dec 4;27(1):18–28. doi: 10.1111/plb.13747 (PMC11656286; doi:10.1111/plb.13747)

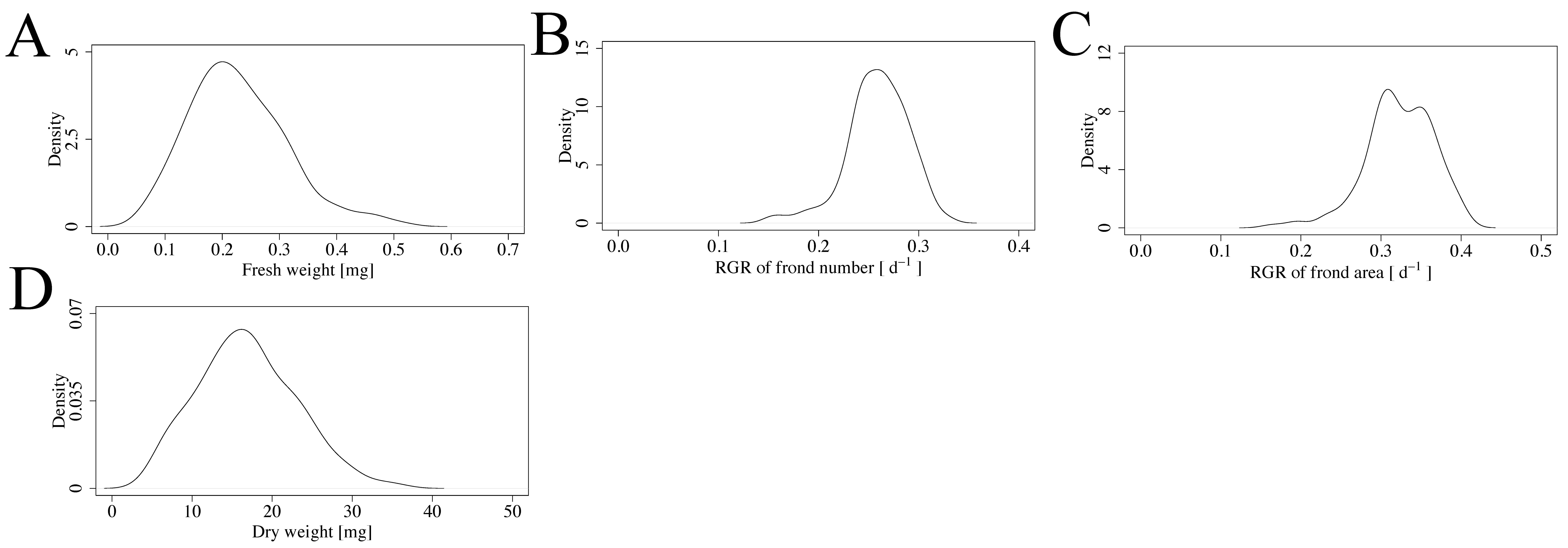

Supplement: Supplementary file 1 — Figure S1. Kernel density distribution of growth and biomass parameters measured for 137 genotypes of S. polyrhiza: Fresh weight (A), RGR of frond number (B), RGR of frond area (C) and dry weight (D) for 137 genotypes of S. polyrhiza. [file PLB-27-18-s006.jpg]

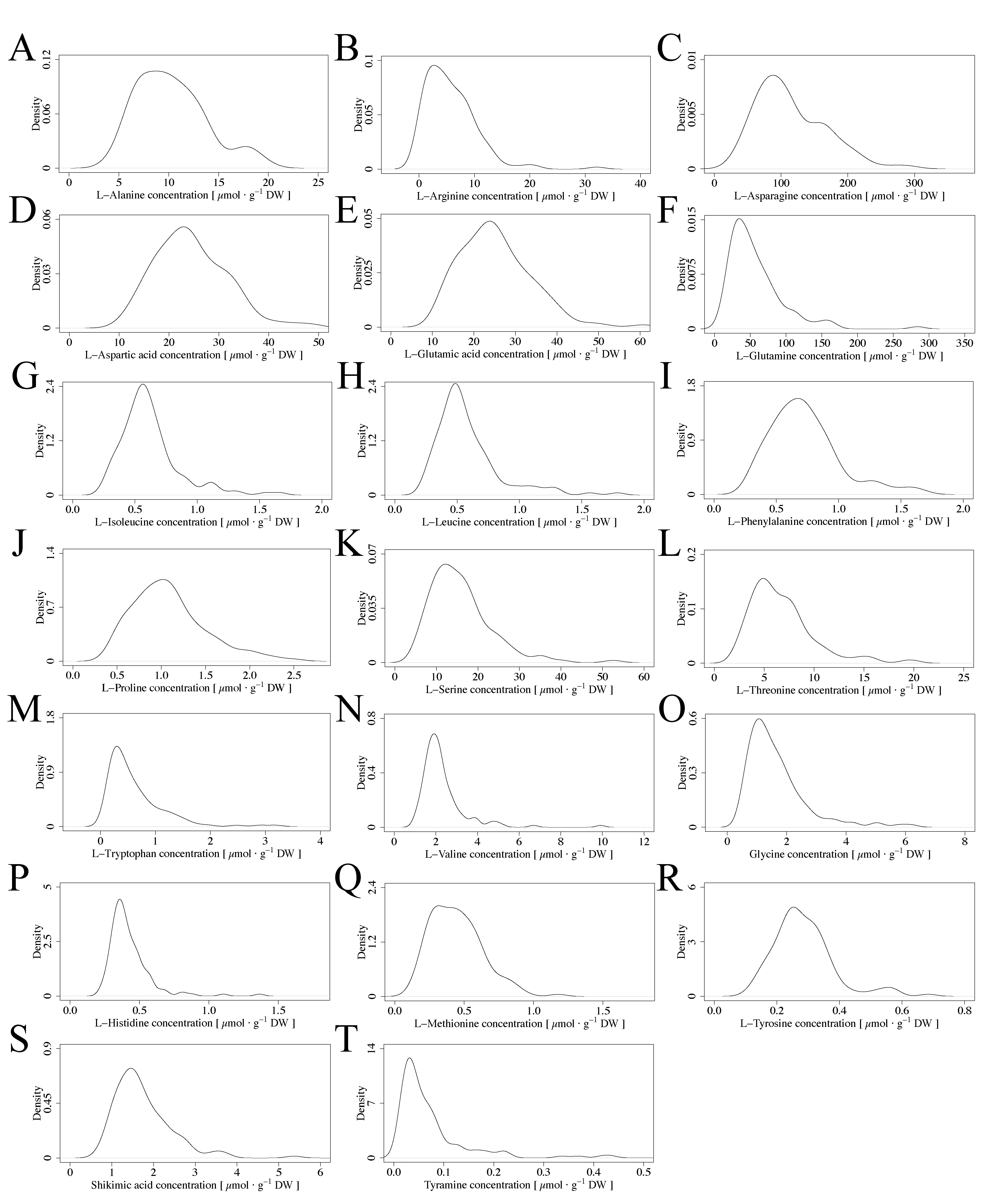

Supplement: Supplementary file 2 — Figure S2. Kernel density distribution of free amino acid concentrations measured for 137 genotypes of S. polyrhiza: L‐Alanine (A), L‐Arginine (B), L‐Asparagine (C), L‐Aspartic acid (D), L‐Glutamic acid (E), L‐Glutamine (F), L‐Isoleucine (G), L‐Leucine (H), L‐Phenylalanine (I), L‐Proline (J), L‐Serine (K), L‐Threonine (L), L‐Tryptophan (M), L‐Valine (N), Glycine (O), L‐Histidine (P), L‐Methionine (Q), L‐Tyrosine (R), Shikimic acid (S) and Tyramine (T). [file PLB-27-18-s001.jpg]

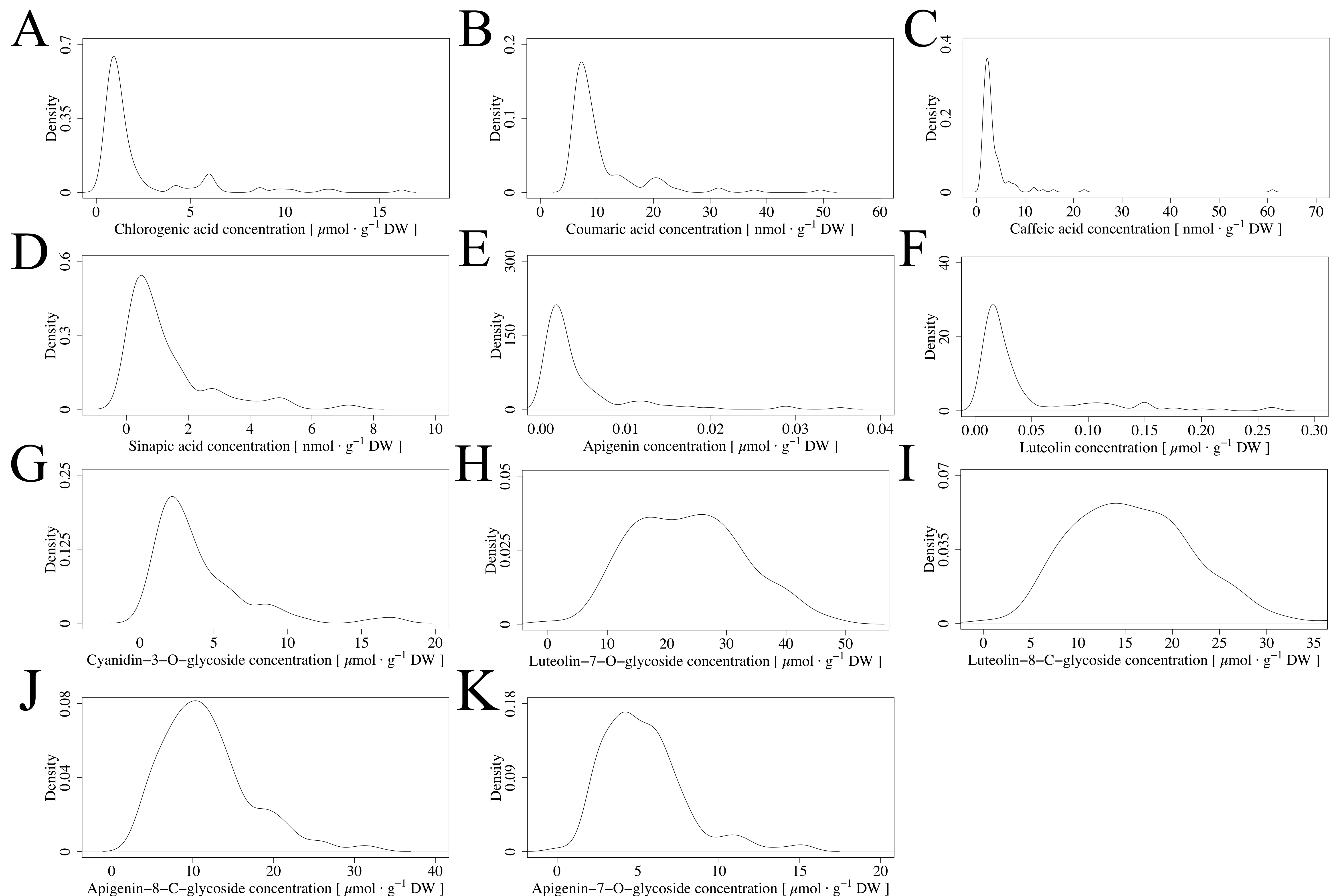

Supplement: Supplementary file 3 — Figure S3. Kernel density distribution of secondary metabolite concentrations measured for 137 genotypes of S. polyrhiza: Chlorogenic acid (A), Coumaric acid (B), Caffeic acid (C), Sinapic acid (D), Apigenin (E), Luteolin (F), Cyanidin‐3‐O‐glycoside (G), Luteolin‐7‐O‐glycoside (H), Luteolin‐8‐C‐glycoside (I), Apigenin‐8‐C‐glycoside (J) and Apigenin‐7‐O‐glycoside (K). [file PLB-27-18-s008.tif]

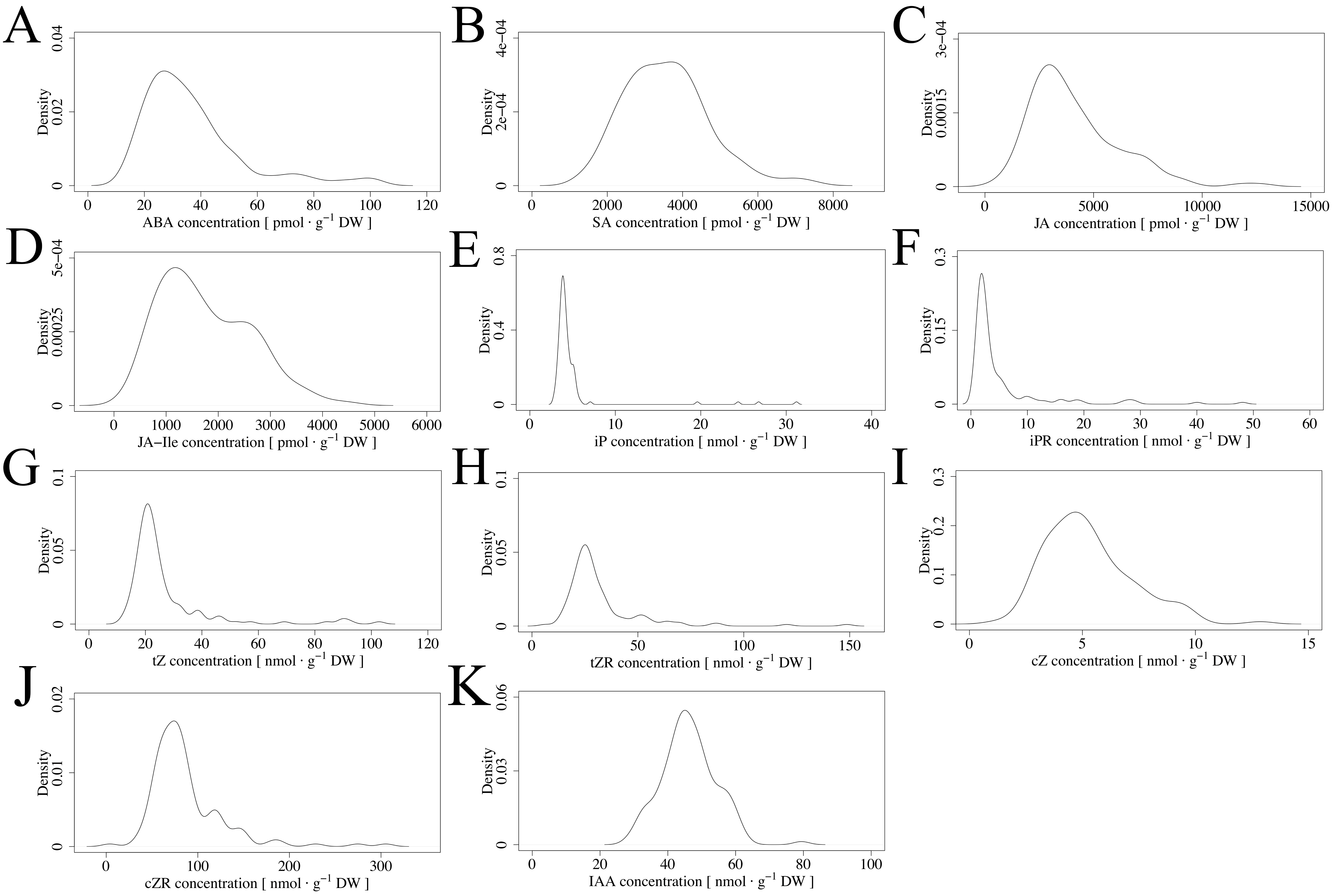

Supplement: Supplementary file 4 — Figure S4. Kernel density distribution of phytohormone concentrations measured for 137 genotypes of S. polyrhiza: Abscisic acid (A), Salicylic acid (B), Jasmonic acid (C), Jasmonic acid‐Isoleucine conjugate (D), N6–Isopentenyladenine (E), N6–Isopentenyladenine riboside (F), trans‐Zeatin (G), trans‐Zeatin riboside (H), cis‐Zeatin (I), cis‐Zeatin riboside (J), Indole‐3‐acetic acid (K). [file PLB-27-18-s004.tif]

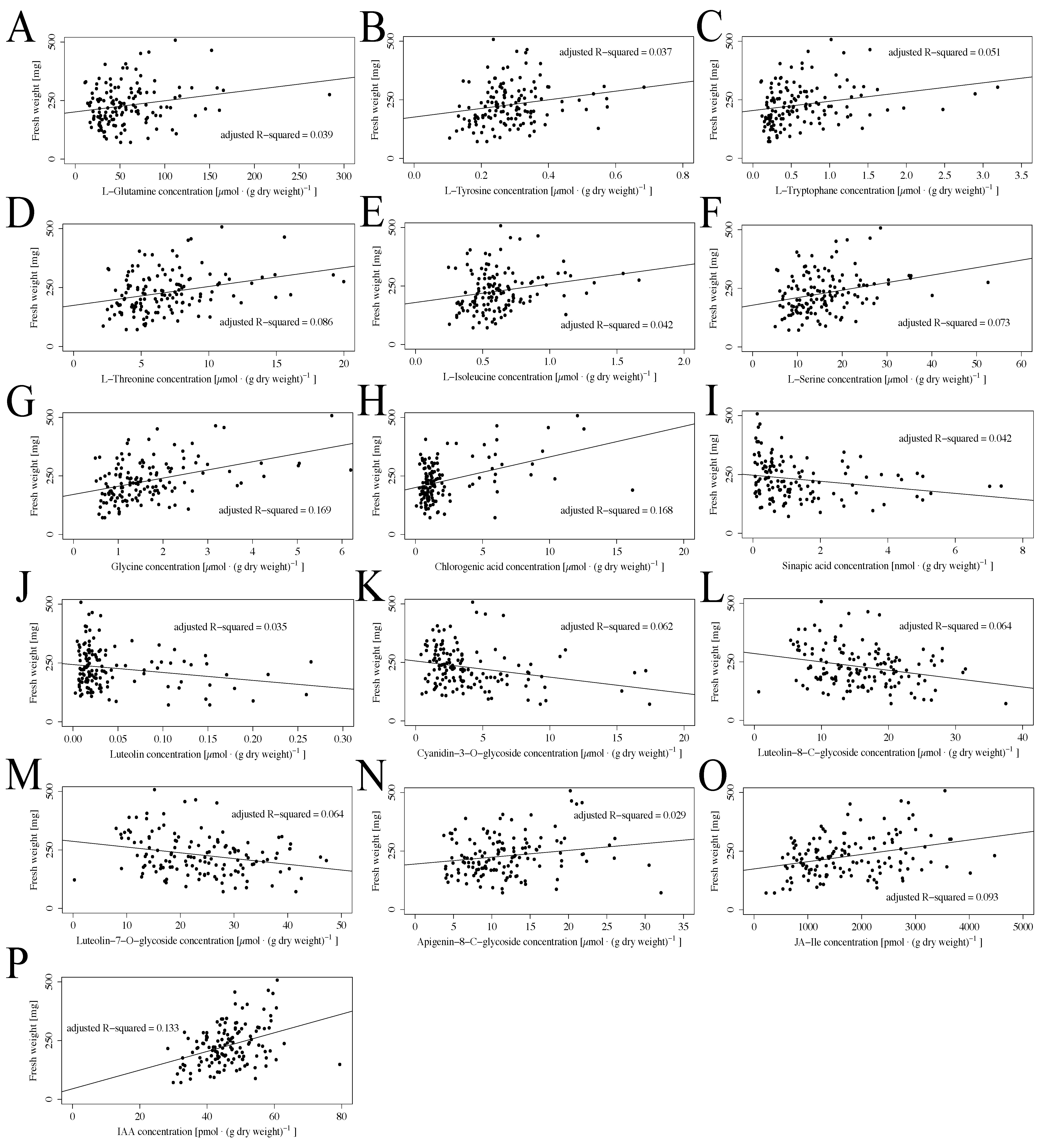

Supplement: Supplementary file 5 — Figure S5. Scatterplots of all significant correlations (F‐test, P ≤ 0.05) of fresh weight with individual free metabolite contents. Fresh weight significantly correlated with contents of L‐Glutamine (A), L‐Tyrosine (B), L‐Tryptophane (C), L‐Threonine (D), L‐Isoleucine (E), L‐Serine (F), Glycine (G), Chlorogenic acid (H), Sinapic acid (I), Luteolin (J), Cyanidin‐3‐O‐glycoside (K), Luteolin‐8‐C‐glycoside (L), Luteolin‐7‐O‐glycoside (M), Apigenin‐8‐C‐glycoside (N), Jasmonic acid–Isoleucine (O) and Indole‐3‐acetic acid (P). [file PLB-27-18-s003.jpg]

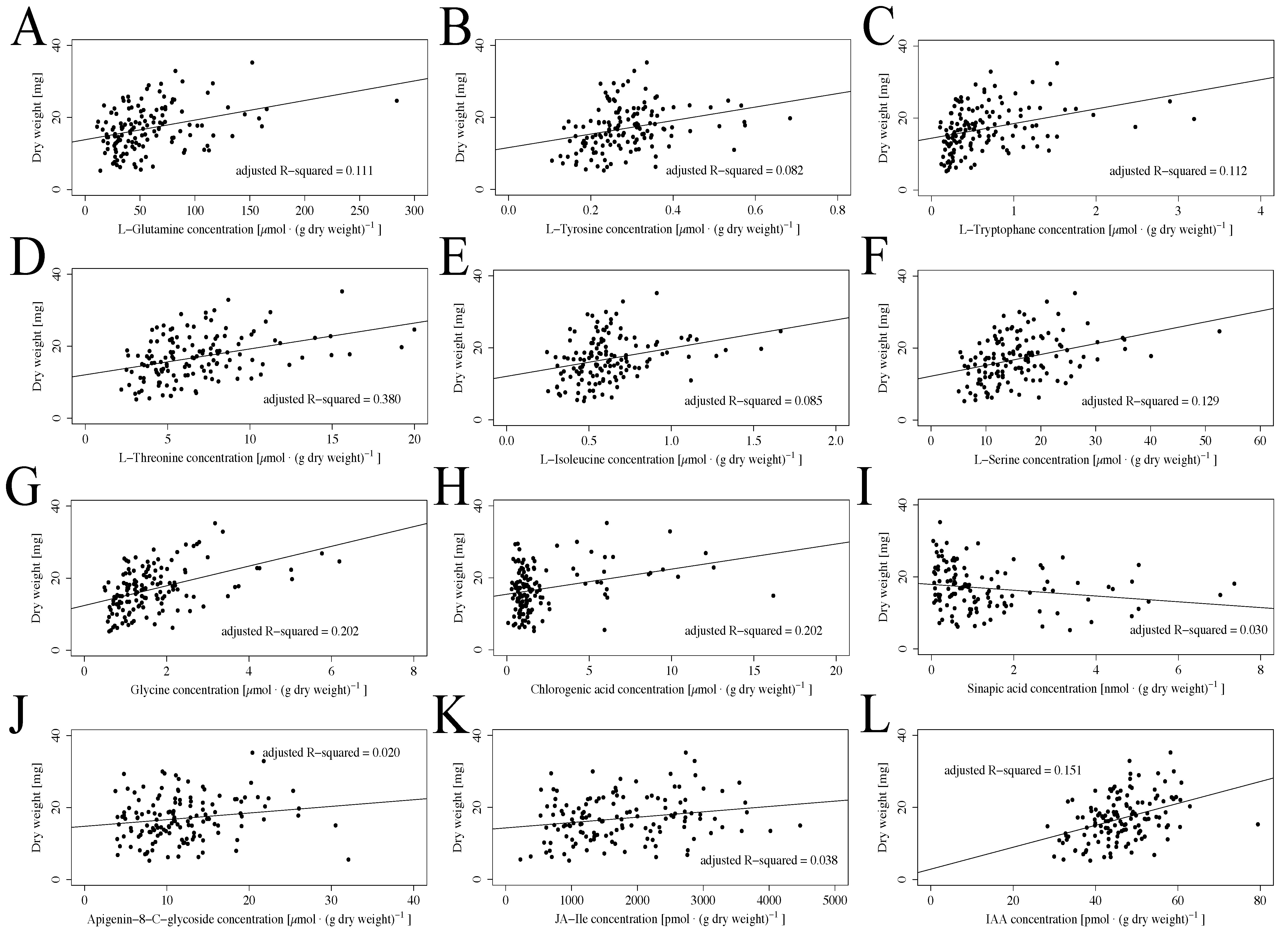

Supplement: Supplementary file 6 — Figure S6. Scatterplots showing all identified significant correlations (F‐test, P ≤ 0.05) of individual free metabolite contents with dry weight. Dry weight was significantly correlated with levels of L‐Glutamine (A), L‐Tyrosine (B), L‐Tryptophane (C), L‐Threonine (D), L‐Isoleucine (E), L‐Serine (F), Glycine (G), Chlorogenic acid (H), Sinapic acid (I), Apigenin‐8‐C‐glycoside (J), Jasmonic acid–Isoleucine (K), Indole‐3‐acetic acid (L). [file PLB-27-18-s002.jpg]

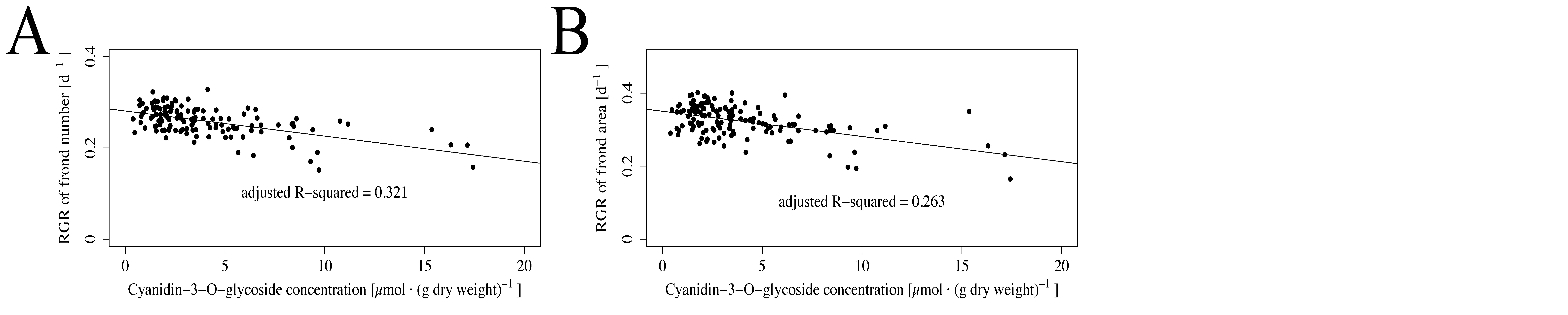

Supplement: Supplementary file 7 — Figure S7. Correlation of growth with contents of Cyanidin‐3‐O‐glycoside. Both metabolites showed strong negative correlation patterns with RGR of frond number (Pearson, ρ = −0.57) (A) and RGR of frond area (Pearson, ρ = −0.52) (B). [file PLB-27-18-s007.jpg]

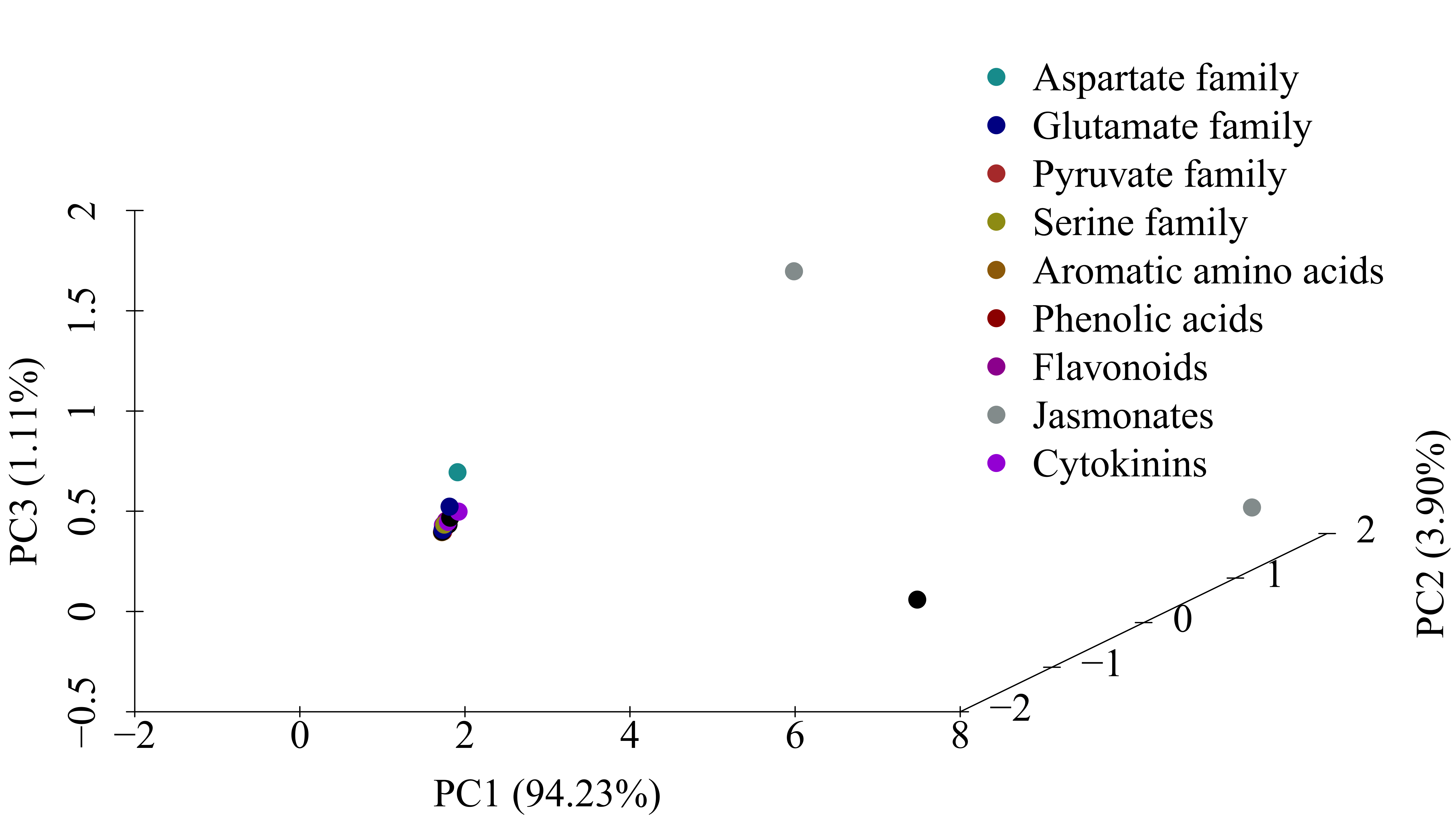

Supplement: Supplementary file 8 — Figure S8. Principal coordinate analysis (PCA) on levels of 42 metabolites, that were categorized into nine groups according to their biosynthetic origin. Contents of all free metabolites except that of salicylic acid (SA) and Jasmonates (JA and JA‐Ile) formed a cluster. [file PLB-27-18-s009.tif]
