# Supplementary material for: Genome‐wide association study of metabolic traits in the giant duckweed Spirodela polyrhiza
Source: Plant Biol (Stuttg). 2024 Dec 4;27(1):18–28. doi: 10.1111/plb.13747 (PMC11656286; doi:10.1111/plb.13747)
Supplement: Supplementary file 10 — Data S2. Supplementary Tables. [file PLB-27-18-s010.docx]

**Supplementary tables**

Table S1: List of all measured metabolites with their metabolic classification. The numbers listed in the Method column correspond to the quantification protocols listed in the Supplemental methods part.

| Metabolite | Class | Method |
| --- | --- | --- |
| L-Alanine | Amino acid metabolism | 1 |
| L-Arginine | Amino acid metabolism | 1 |
| L-Asparagine | Amino acid metabolism | 1 |
| L-Aspartic acid | Amino acid metabolism | 1 |
| L-Glutamic acid | Amino acid metabolism | 1 |
| L-Glutamine | Amino acid metabolism | 1 |
| L-Isoleucine | Amino acid metabolism | 1 |
| L-Leucine | Amino acid metabolism | 1 |
| L-Phenylalanine | Amino acid metabolism | 1 |
| L-Proline | Amino acid metabolism | 1 |
| L-Serine | Amino acid metabolism | 1 |
| L-Threonine | Amino acid metabolism | 1 |
| L-Tryptophan | Amino acid metabolism | 1 |
| L-Valine | Amino acid metabolism | 1 |
| Chlorogenic acid | Specialized metabolites | 1 |
| Cyanidin-3-O-glycoside | Specialized metabolites | 1 |
| Luteolin-8-C-glycoside | Specialized metabolites | 1 |
| Luteolin-7-O-glycoside | Specialized metabolites | 1 |
| Apigenin-8-C-glycoside | Specialized metabolites | 1 |
| Apigenin-7-O-glycoside | Specialized metabolites | 1 |
| Glycine | Amino acid metabolism | 2 |
| L-Histidine | Amino acid metabolism | 2 |
| L-Methionine | Amino acid metabolism | 2 |
| L-Tyrosine | Amino acid metabolism | 2 |
| Tyramine | Amino acid metabolism | 2 |
| Shikimic acid | Amino acid metabolism | 2 |
| Apigenin | Specialized metabolites | 2 |
| Luteolin | Specialized metabolites | 2 |
| ABA | Phytohormones | 3 |
| SA | Phytohormones | 3 |
| JA | Phytohormones | 3 |
| JA-Ile | Phytohormones | 3 |
| Coumaric acid | Specialized metabolites | 4 |
| Caffeic acid | Specialized metabolites | 4 |
| Sinapic acid | Specialized metabolites | 4 |
| IAA | Phytohormones | 4 |
| iP | Phytohormones | 5 |
| iPR | Phytohormones | 5 |
| tZ | Phytohormones | 5 |
| tZR | Phytohormones | 5 |
| cZ | Phytohormones | 5 |
| cZR | Phytohormones | 5 |

Abbreviations: ABA – Abscisic acid, cZ – cis-Zeatin, cZR – cis-Zeatin riboside, IAA – Indoleacetic acid, iP – Isopentenyladenine, iPR – Isopentenyladenine riboside, JA – Jasmonic acid, JA-Ile – Jasmonic acid-isoleucine conjugate, SA – Salicylic acid, tZ – trans-Zeatin, tZR – trans-Zeatin riboside

Table S2: Technical parameters used for quantification of the amino acids and secondary metabolites quantified with Method S1 via LC-MS.

| Metabolite | Ionization mode | Start [min] | End [min] | Q1 [m/z] | Q3 [m/z] | Dwell time [ms] | CE | Q1/Q3 Pre Bias [V] | |
| --- | --- | --- | --- | --- | --- | --- | --- | --- | --- |
| L-Alanine | + | 0.20 | 0.85 | 90.05 | 44.20 | 20 | -13 | -10/-18 | |
| L-Arginine | + | 0.20 | 0.85 | 175.12 | 60.20 | 20 | -14 | -20/-24 | |
| L-Aspartate | + | 0.20 | 0.85 | 134.04 | 88.20 | 20 | -12 | -13/-16 | |
| L-Glutamate | + | 0.20 | 0.85 | 148.06 | 102.15 | 20 | -13 | -10/-19 | |
| [^13^C_3_, ^15^N_1_]-Alanine | + | 0.20 | 0.85 | 94.06 | 47.20 | 20 | -13 | -10/-18 | |
| [^13^C_6_, ^15^N_4_]-Arginine | + | 0.20 | 0.85 | 185.13 | 64.15 | 20 | -14 | -20/-24 | |
| [^13^C_4_, ^15^N_n_]-Aspartate | + | 0.20 | 0.85 | 139.06 | 92.25 | 20 | -12 | -13/-16 | |
| [^13^C_5_, ^15^N_n_]-Glutamate | + | 0.20 | 0.85 | 154.07 | 107.20 | 20 | -13 | -10/-19 | |
| [^13^C_5_, ^15^N_1_]-Proline | + | 0.20 | 0.85 | 122.08 | 75.20 | 20 | -17 | -11/-20 | |
| [^13^C_3_, ^15^N_1_]-Serine | + | 0.20 | 0.85 | 110.06 | 63.20 | 20 | -14 | -10/-23 | |
| [^13^C_4_, ^15^N_1_]-Threonine | + | 0.20 | 0.85 | 125.08 | 78.20 | 20 | -12 | -12/-30 | |
| L-Proline | + | 0.20 | 0.85 | 116.07 | 70.20 | 20 | -17 | -11/-20 | |
| L-Serine | + | 0.20 | 0.85 | 106.05 | 60.20 | 20 | -14 | -10/-23 | |
| L-Threonine | + | 0.20 | 0.85 | 120.07 | 74.20 | 20 | -12 | -12/-30 | |
| L-Asparagine | + | 0.20 | 0.85 | 133.06 | 87.20 | 20 | -11 | -13/-16 | |
| L-Glutamine | + | 0.20 | 0.85 | 147.08 | 130.15 | 20 | -15 | -10/-13 | |
| [^13^C_5_, ^15^N_1_]-Valine | + | 0.35 | 1.05 | 124.10 | 77.20 | 20 | -12 | -22/-28 | |
| L-Valine | + | 0.35 | 1.05 | 118.09 | 72.20 | 20 | -12 | -22/-28 | |
| L-Isoleucine | + | 0.85 | 1.70 | 132.10 | 86.15 | 122 | -11 | -20/-20 | |
| [^13^C_6_, ^15^N_1_]-Isoleucine | + | 0.85 | 1.70 | 139.12 | 92.25 | 122 | -11 | -20/-20 | |
| L-Leucine | + | 0.85 | 1.70 | 132.10 | 86.15 | 122 | -11 | -20/-20 | |
| [^13^C_6_, ^15^N_1_]-Leucine | + | 0.85 | 1.70 | 139.12 | 92.25 | 122 | -11 | -20/-20 | |
| [^13^C_9_, ^15^N_1_]-Phenylalanine | + | 2.00 | 3.00 | 176.11 | 129.25 | 247 | -15 | -20/-20 |  |
| L-Phenylalanine | + | 2.00 | 3.00 | 166.09 | 120.20 | 247 | -15 | -20/-20 | |
| Cyanidin-3-O-glycoside | + | 3.00 | 3.70 | 449.10 | 137.10 | 32 | -54 | -20/-14 | |
| Chlorogenic acid | + | 3.00 | 3.70 | 355.10 | 163.15 | 32 | -15 | -23/-17 | |
| L-Tryptophan | + | 3.00 | 3.70 | 205.10 | 188.10 | 32 | -10 | -20/-20 | |
| Apigenin-7-O-glycoside | + | 3.40 | 4.10 | 431.10 | 268.10 | 32 | -35 | -21/-17 |  |
| Apigenin-8-C-glycoside | + | 3.40 | 4.10 | 431.10 | 311.20 | 32 | -24 | -30/-21 |  |
| Luteolin-7-O-glycoside | + | 3.40 | 4.10 | 447.09 | 285.20 | 32 | -26 | -22/-19 |  |
| Luteolin-8-C-glycoside | + | 3.40 | 4.10 | 447.09 | 327.15 | 32 | -24 | -30/-22 |  |

Abbreviations: CE - Collision energy

Table S3: Technical parameters used for quantification of the amino acids and secondary metabolites with Method S2 via LC-MS.

| Metabolite | Ionization mode | Start [min] | End [min] | Q1 [m/z] | Q3 [m/z] | Dwell time [ms] | CE | Q1/Q3 Pre Bias [V] |
| --- | --- | --- | --- | --- | --- | --- | --- | --- |
| Glycine | + | 0.20 | 0.90 | 76.04 | 30.20 | 50 | -12 | -14/-11 |
| [^13^C_2_, ^15^N_1_]-Glycine | + | 0.20 | 0.90 | 79.04 | 32.10 | 50 | -12 | -14/-11 |
| L-Histidine | + | 0.20 | 1.00 | 156.08 | 110.20 | 50 | -15 | -30/-21 |
| [^13^C_6_, ^15^N_3_]-Histidine | + | 0.20 | 1.00 | 165.09 | 118.20 | 50 | -15 | -30/-21 |
| [^13^C_5_, ^15^N_1_]-Methionine | + | 0.40 | 1.10 | 156.07 | 63.15 | 50 | -22 | -10/-24 |
| L-Methionine | + | 0.40 | 1.10 | 150.06 | 61.16 | 50 | -22 | -10/-24 |
| Shikimic acid | - | 0.40 | 1.10 | 173.05 | 93.10 | 23 | 15 | 12/21 |
| [^13^C_9_, ^15^N_1_]-Tyrosine | + | 0.40 | 2.00 | 192.11 | 145.20 | 50 | -15 | -18/-14 |
| L-Tyrosine | + | 0.40 | 2.00 | 182.08 | 136.20 | 50 | -15 | -18/-14 |
| Tyramine | + | 0.40 | 2.00 | 138.09 | 121.15 | 23 | -15 | -20/-20 |
| Apigenin | - | 3.80 | 4.50 | 269.05 | 151.20 | 147 | 25 | 30/14 |
| Luteolin | - | 3.80 | 4.50 | 285.04 | 132.20 | 147 | 48 | 30/26 |

Abbreviations: CE - collision energy

Table S4: Technical parameters used for quantification of metabolites with Method S3 via LC-MS.

| Metabolite | Ionization mode | Start [min] | End [min] | Q1 [m/z] | Q3 [m/z] | Dwell time [ms] | CE | Q1/Q3 Pre Bias [V] | |
| --- | --- | --- | --- | --- | --- | --- | --- | --- | --- |
| SA | - | 2.20 | 3.20 | 137.02 | 92.95 | 20 | 19 | 27/19 | |
| [^2^H_4_]-SA | - | 2.20 | 3.20 | 141.05 | 97.00 | 20 | 19 | 27/15 |  |
| ABA | - | 2.20 | 3.20 | 263.13 | 153.10 | 20 | 13 | 18/29 |  |
| [^2^H_6_]-ABA | - | 2.20 | 3.20 | 269.17 | 159.20 | 20 | 13 | 18/30 |  |
| JA | - | 2.75 | 3.75 | 209.12 | 59.00 | 20 | 14 | 14/11 |  |
| [^2^H_5_]-JA | - | 2.75 | 3.75 | 214.15 | 62.00 | 20 | 14 | 14/10 |  |
| JA-Ile | - | 3.75 | 5.25 | 322.20 | 130.10 | 135 | 22 | 24/20 |  |

Abbreviations: ABA – Abscisic acid, CE - collision energy, JA – Jasmonic acid, JA-Ile – Jasmonic acid-isoleucine conjugate, SA – Salicylic acid

Table S5: Technical parameters used for quantification of the metabolites with Method S4 via LC-MS.

| Metabolite | Ionization mode | Start [min] | End [min] | Q1 [m/z] | Q3 [m/z] | Dwell time [ms] | CE | Q1/Q3 Pre Bias [V] |
| --- | --- | --- | --- | --- | --- | --- | --- | --- |
| Caffeic acid | - | 1.95 | 2.70 | 179.03 | 135.05 | 20 | 18 | 12/26 |
| Coumaric acid | - | 2.30 | 3.05 | 163.04 | 119.10 | 20 | 17 | 11/11 |
| Sinapic acid | + | 2.45 | 3.15 | 225.08 | 207.15 | 20 | -9 | -15/-23 |
| IAA | + | 2.90 | 3.65 | 176.07 | 130.10 | 20 | -16 | -12/-23 |
| [^13^C_6_]-IAA | + | 2.90 | 3.65 | 182.09 | 136.25 | 20 | -16 | -12/-13 |

Abbreviations: CE - collision energy, IAA – Indole-3-acetic acid

Table S6: Technical parameters used for quantification of the metabolites with Method S5 via LC-MS.

| Metabolite | Ionization mode | Start [min] | End [min] | Q1 [m/z] | Q3 [m/z] | Dwell time [ms] | CE | Q1/Q3 Pre Bias [V] |
| --- | --- | --- | --- | --- | --- | --- | --- | --- |
| [^2^H_5_]-tZ | + | 2.00 | 3.00 | 225.15 | 137.20 | 47 | -20 | -21/-22 |
| tZ | + | 2.00 | 3.50 | 220.12 | 136.10 | 47 | -20 | -20/-20 |
| cZ | + | 2.00 | 3.50 | 220.12 | 136.10 | 47 | -20 | -20/-20 |
| tZR | + | 3.75 | 5.25 | 352.16 | 220.15 | 30 | -20 | -20/-20 |
| cZR | + | 3.75 | 5.25 | 352.16 | 220.15 | 30 | -20 | -20/-20 |
| iP | + | 5.00 | 6.00 | 204.12 | 136.10 | 30 | -15 | -20/-20 |
| [^2^H_6_]-iP | + | 5.00 | 6.00 | 210.16 | 137.20 | 30 | -15 | -20/-20 |
| iPR | + | 6.00 | 7.00 | 336.17 | 204.20 | 47 | -20 | -20/-20 |
| [^2^H_6_]-iPR | + | 6.00 | 7.00 | 342.20 | 210.25 | 47 | -20 | -20/-20 |

Abbreviations: CE – Collision energy, cZ – cis-Zeatin, cZR – cis-Zeatin riboside, iP – Isopentenyladenine, iPR -Isopentenyladenine riboside, tZ – trans-Zeatin, tZR – trans-Zeatin riboside

Table S7: Primers used for RT-qPCR analysis of *SpUBP7* and reference gene (*SpaEF* and *SpGAPDH*) expression. The primers for the reference genes were used in previous publications (Wang et al. 2024; Höfer et al. 2024b).

| Gene | Gene ID | Forward Primer (5’-3’) | Reverse Primer (5’-3’) | Product size | Efficiency |
| --- | --- | --- | --- | --- | --- |
| *SpGAPDH* | *SpGA2022_054082* | AGCATCCAAGAAGGTGAAGATCGGC | TTGTAGTCGGTCGTGATGAAGGGG | 132 bp | 104.4% |
| *SpaEF* | *SpGA2022_005771* | TCGAAGCCGGCATTTCCAAGGACG | TCGCCTTCGAGTACTTGGGTGTCG | 129 bp | 99.8% |
| *SpUBP7* | *SpGA2022_056000* | GACGCAGATTGGTCAACCTTAGGTG | CCGGAACTGAATGTAGACACTGGATTG | 217 bp | 101.7% |

Table S8: Variation of compounds belonging to amino acid metabolism in 137 *S. polyrhiza* genotypes.

| Metabolite | Mean [µmol/g DW] | SE [µmol/g DW] | Range [µmol/g DW]  Minimum Maximum | | Fold change | H2.c |
| --- | --- | --- | --- | --- | --- | --- |
| Glycine | 1.650 | 0.087 | 0.488 | 6.193 | 12.691 | 0.701 |
| L-Serine | 15.775 | 0.638 | 5.034 | 52.559 | 10.441 | 5.706 · 10^-9^ |
| L-Alanine | 10.283 | 0.301 | 3.746 | 20.019 | 5.344 | 0.026 |
| L-Valine | 2.298 | 0.094 | 1.003 | 9.915 | 9.885 | 0.572 |
| L-Leucine | 0.579 | 0.022 | 0.232 | 1.786 | 7.698 | 0.771 |
| L-Aspartate | 24.468 | 0.622 | 10.529 | 49.306 | 4.683 | 1.336 · 10^-10^ |
| L-Asparagine | 114.414 | 4.459 | 29.514 | 293.244 | 9.936 | 2.220 · 10^-15^ |
| L-Methionine | 0.446 | 0.016 | 0.118 | 1.177 | 9.975 | 0.027 |
| L-Threonine | 6.759 | 0.276 | 2.187 | 19.990 | 9.140 | 0.187 |
| L-Isoleucine | 0.616 | 0.020 | 0.245 | 1.664 | 6.792 | 0.350 |
| L-Glutamine | 57.034 | 3.280 | 10.845 | 283.849 | 26.173 | 0.348 |
| L-Glutamate | 25.204 | 0.726 | 11.234 | 60.675 | 5.401 | 5.477 · 10^-10^ |
| L-Arginine | 5.727 | 0.401 | 0.293 | 32.078 | 109.481 | 0.161 |
| L-Proline | 1.089 | 0.036 | 0.407 | 2.505 | 6.155 | 0.087 |
| L-Phenylalanine | 0.729 | 0.024 | 0.281 | 1.675 | 5.961 | 0.315 |
| L-Tyrosine | 0.285 | 0.008 | 0.106 | 0.684 | 6.453 | 0.591 |
| L-Tryptophan | 0.623 | 0.044 | 0.104 | 3.190 | 30.673 | 0.372 |
| Tyramine | 0.076 | 0.007 | 0.013 | 0.623 | 47.923 | 0.810 |
| Shikimic acid | 1.798 | 0.080 | 0.695 | 9.173 | 13.199 | 0.732 |

Abbreviations: SE- standard error, H2.c – broad sense heritability according to Cullis et al. (2006)

Table S9: Variation of phytohormone concentrations in 137 *S. polyrhiza* genotypes.

| Metabolite | Mean [nmol/g DW] | SE [nmol/g DW] | Range [nmol/gDW] Minimum Maximum | | Fold change | H2.c |
| --- | --- | --- | --- | --- | --- | --- |
| SA | 3.585 | 0.098 | 1.295 | 7.424 | 5.733 | 0.373 |
| ABA | 0.037 | 0.002 | 0.014 | 0.102 | 7.286 | 2.327 · 10^-7^ |
| JA | 4.147 | 0.180 | 0.547 | 12.726 | 23.265 | 0.414 |
| JA-Ile | 1.760 | 0.074 | 0.223 | 4.471 | 20.049 | 0.382 |
| iP | 4.731 | 0.317 | 2.847 | 31.200 | 10.959 | 0.999 |
| iPR | 5.121 | 0.624 | 1.012 | 48.089 | 47.519 | 0.709 |
| tZ | 26.715 | 1.310 | 11.813 | 102.724 | 8.696 | 0.473 |
| tZR | 31.930 | 1.573 | 6.209 | 148.603 | 23.933 | 0.349 |
| cZ | 5.268 | 0.165 | 1.212 | 12.868 | 10.617 | 0.333 |
| cZR | 86.971 | 3.509 | 4.016 | 305.175 | 75.990 | 0.424 |
| IAA | 46.315 | 0.683 | 28.333 | 79.422 | 2.803 | 0.180 |

Abbreviations: ABA – Abscisic acid, cZ – cis-Zeatin, cZR – cis-Zeatin riboside, IAA – Indoleacetic acid, iP – Isopentenyladenine, iPR – Isopentenyladenine riboside, JA – Jasmonic acid, JA-Ile – Jasmonic acid-isoleucine conjugate, SA – Salicylic acid, tZ – trans-Zeatin, tZR – trans-Zeatin riboside, SE- standard error, H2.c – broad sense heritability according to Cullis et al. (2006)

Table S10: Variation of secondary metabolites concentrations in 137 *S. polyrhiza* genotypes.

| Metabolite | Mean [nmol/g DW] | SE [nmol/g DW] | Range [nmol/g DW] Minimum Maximum | | Fold change | | H2.c |
| --- | --- | --- | --- | --- | --- | --- | --- |
| Chlorogenic acid | 2147.696 | 230.439 | 75.289 | 16181.370 | | 214.923 | 0.979 |
| Coumaric acid | 10.461 | 0.555 | 5.078 | 49.489 | | 9.746 | 0.529 |
| Caffeic acid | 3.784 | 0.485 | 1.029 | 60.979 | | 59.260 | 0.927 |
| Sinapic acid | 1.318 | 0.128 | 0.044 | 7.361 | | 167.295 | 0.839 |
| Apigenin | 2.361 | 0.473 | 0.420 | 35.256 | | 93.943 | 0.045 |
| Luteolin | 42.592 | 4.427 | 2.666 | 264.595 | | 98.498 | 0.440 |
| Cyanidin-3-O-glycoside | 3972.074 | 273.743 | 403.464 | 17431.470 | | 43.205 | 0.593 |
| Luteolin-8-C-glycoside | 15861.540 | 539.504 | 649.239 | 37498.920 | | 57.758 | 0.112 |
| Luteolin-7-O-glycoside | 23596.070 | 780.300 | 256.907 | 47213.030 | | 183.775 | 0.057 |
| Apigenin-8-C-glycoside | 11698.560 | 471.242 | 3696.448 | 32069.280 | | 8.676 | 0.015 |
| Apigenin-7-O-glycoside | 5348.446 | 222.837 | 110.307 | 15238.690 | | 138.148 | 1.294 · 10^-10^ |

Abbreviations: SE- standard error, H2.c – broad sense heritability according to Cullis et al. (2006)

Table S11: Number of SVs and SNPs significantly associated with the corresponding parameters (Bonferroni-corrected *P*-Wald < 0.05). Parameters with no significant associations were not listed.

| Parameter | Number significant markers-SVs | Number significant markers-SNPs |
| --- | --- | --- |
| RGR frond area | 1 | 0 |
| L-Arginine | 1 | 1 |
| L-Glutamine | 1 | 2 |
| L-Histidine | 1 | 3 |
| L-Leucine | 0 | 1 |
| L-Serine | 1 | 0 |
| L-Threonine | 0 | 1 |
| L-Tryptophan | 0 | 3 |
| L-Tyrosine | 0 | 6 |
| L-Valine | 0 | 2 |
| Shikimic acid | 0 | 7 |
| Tyramine | 0 | 3 |
| Chlorogenic acid | 1 | 5 |
| Coumaric acid | 1 | 1 |
| Caffeic acid | 1 | 0 |
| Sinapic acid | 1 | 2 |
| Luteolin | 1 | 1 |
| Apigenin | 1 | 2 |
| Cyanidin-3-O-glycoside | 0 | 1 |
| iP | 3 | 12 |
| iPR | 0 | 7 |
| tZ | 1 | 6 |
| tZR | 0 | 6 |
| cZ | 1 | 0 |
| cZR | 0 | 1 |
| IAA | 0 | 2 |

Abbreviations: ABA – Abscisic acid, cZ – cis-Zeatin, cZR – cis-Zeatin riboside, IAA – Indoleacetic acid, iP – Isopentenyladenine, iPR – Isopentenyladenine riboside, JA – Jasmonic acid, JA-Ile – Jasmonic acid-isoleucine conjugate, SA – Salicylic acid, tZ – trans-Zeatin, tZR – trans-Zeatin riboside

Table S12: SVs significantly associated with metabolome and fitness data. The significance threshold was set to 4.23·10^-5^ (Bonferroni corrected *P*-Wald value of 0.05 for 1182 markers).

| Metabolite | Chromosome | Position | Associated gene locus and description | Location | Type of SV | | Size of SV | *P*-Wald |
| --- | --- | --- | --- | --- | --- | --- | --- | --- |
| L-Arginine | 16 | 3514401 | - | Intergenic region: > 30 kbp away from next coding sequence | | insertion | 56 bp | 1.32·10^-6^ |
| L-Glutamine, L-Serine | 19 | 51003 | *SpGA2022_056000*: *SpUBP7* (Ubiquitin-specific Protease 7) | Intronic region (7th intron) of *SpUBP7* | | deletion | 94 bp | 1.48·10^-5^, 1.75·10^-5^ |
| L-Histidine | 1 | 2805097 | *SpGA2022_002723* | 1814 bp upstream of SpGA2022_002723 | | insertion | 97 bp | 6.44·10^-8^ |
| L-Leucine | 14 | 4059780 | *SpGA2022_017710*: *SpROC1* (Homeobox-leucine zipper protein R) | 448 bp upstream of SpROC1 | | deletion | 75 bp | 2.96·10^-5^ |
| Chlorogenic acid | 10 | 421049 | *SpGA2022_013652* | 12392 bp upstream of *SpGA2022_013652* | | deletion | 54 bp | 7.33·10^-7^ |
| Caffeic acid | 12 | 4246070 | SpGA2022_016032: similar to At5g10620 (Putative RNA methyltransferase) | Intronic region (6th intron) of SpGA2022_016032 | | duplication | 265 bp | 4.04·10^-5^ |
| Sinapic acid, Apigenin, Luteolin | 16 | 4471839 | - | Intergenic region: > 30 kbp away from next coding sequence | | deletion | 266 bp | 1.29·10^-9^, 4.59·10^-7^, 1.31·10^-6^ |
| iP | 6 | 6329355 | *SpGA2022_009939*: Similar to Protein yippee-like | 10473 bp upstream of *SpGA2022_009939* | | deletion | 54 bp | 2.16·10^-6^ |
| iP | 13 | 2006566 | *SpGA2022_054972*: *SpNAP1* | Intronic region (5th intron) of *SpNAP1* | | deletion | 74 bp | 2.98·10^-5^ |
| iP, tZ | 18 | 95646 | *SpGA2022_019361*: *SpTSC10A* (3-dehydrosphinganine reductase) | 12371 bp upstream of *SpTSC10A* | | deletion | 52 bp | 2.48·10^-8^, 3.27·10^-5^ |
| cZ | 13 | 1506652 | *SpGA2022_054953*: *SpSCAMP3* (Secretory carrier-associated membrane protein 3 | 3' UTR of *SpSCAMP3* | | duplication | 76 bp | 6.73·10^-7^ |
| RGR frond area | 6 | 4804486 | - | Intergenic region: > 30 kbp away from next coding sequence | | insertion | 56 bp | 9.11·10^-6^ |

Abbreviations: ABA – Abscisic acid, cZ – cis-Zeatin, cZR – cis-Zeatin riboside, IAA – Indole-3-acetic acid, iP – Isopentenyladenine, iPR – Isopentenyladenine riboside, JA – Jasmonic acid, JA-Ile – Jasmonic acid-isoleucine conjugate, SA – Salicylic acid, tZ – trans-Zeatin, tZR – trans-Zeatin riboside

Table S13: SNPs significantly associated with metabolome traits. The significance threshold was set to 1.14 ·10^-6^ (Bonferroni corrected *P*-Wald value of 0.05 for 10057 markers).

| Parameter | Chromosome | Position | Alleles | Associated gene locus | Function | Location | *P*-Wald |
| --- | --- | --- | --- | --- | --- | --- | --- |
| L-Arginine | 12 | 1022717 | C/T | *SpGA2022_015481*: *SpNAT7* | Nucleobase ascorbate transporter 7 | Intronic region | 2.44·10^-8^ |
| L-Glutamine | 2 | 5658111 | G/A | *SpGA2022_005065* | Unknown function | 2691 bp upstream | 4.08·10^-7^ |
| L-Glutamine  L-Valine | 4 | 6512246 | C/G | *SpGA2022_007596: SpLHCB5* | Light-harvesting complex of photosystem II 5 | Exonic region | 1.63·10^-7^  8.09·10^-7^ |
| L-Histidine | 9 | 2006410 | G/A | *SpGA2022_053849*: *SpCYP71AU50* | Homolog of At1g51810 (Probable receptor-like protein kinase) | 7570 bp upstream | 2.15·10^-9^ |
| L-Histidine | 8 | 2025860 | C/G | *SpGA2022_053495*: *SpPFK3* | ATP-dependent 6-phosphofructokinase 3 | 5334 bp upstream | 3.39·10^-9^ |
| L-Histidine,  L-Tyrosine | 20 | 3499796 | G/A | *SpGA2022_056315*: *SpPUB4* | Plant U-box containing protein 4 | 3404 bp upstream | 6.52·10^-8^, 1.86·10^-8^ |
| L-Tryptophan | 3 | 7646125 | T/A | *SpGA2022_006560* | Unknown function | Exonic region | 7.41·10^-7^ |
| L-Tryptophan  L-Tyrosine  L-Tyramine | 14 | 3558175 | G/T | *SpGA2022_017644* | Unknown function | Intronic region | 7.41·10^-7^  3.52 ·10^-6^  1.39 ·10^-6^ |
| L-Tryptophan  L-Tyrosine  L-Tyramine | 4 | 6512411 | G/A | *SpGA2022_007596*: *SpLHCB5* | Light-harvesting complex of photosystem II 5 | Exonic region | 9.76·10^-7^ |
| L-Tyrosine | 9 | 7058554 | A/G | *SpGA2022_054061*: *SpMYOB7* | Myosin binding protein 7 | 8600 bp upstream | 6.33·10^-7^ |
| L-Tyrosine | 5 | 6307147 | G/A | *SpGA2022_008803*: *SpUGT89B1* | Flavonol 2-O-glycosyltransferase | 13387 bp upstream | 9.23·10^-7^ |
| L-Tyrosine | 3 | 746125 | T/A | *SpGA2022_006560* | Unknown function | Exonic region | 3.52·10^-6^ |
| L-Valine | 1 | 10088583 | G/A | *SpGA2022_003754* | Unknown function | Intronic region | 4.84·10^-8^ |
| Shikimic acid,  iPR,  tZR | 5 | 1506810 | C/G | *SpGA2022_008160*: *SpRTNLB3* | Reticulon-like protein B3 | Exonic region | 7.97·10^-12^, 1.22·10^-14^, 1.36·10^-13^ |
| Shikimic acid,  iPR,  tZR | 2 | 6978275 | G/A | *SpGA2022_005286*: *SpAGL62* | Transcription factor AGAMOUS | 707 bp upstream | 7.97·10^-12^, 1.22·10^-14^, 1.36·10^-13^ |
| Shikimic acid, iP,  iPR  tZ,  tZR | 8 | 2931769 | C/T | *SpGA2022_011897*: *SpLOB* | LOB (Lateral Organ Boundaries) | 12 bp upstream | 1.05·10^-9^, 5.88·10^-19^,  2.19·10^-8^,  8.86·10^-12^,  4.62·10^-8^ |
| Shikimic acid | 1 | 4629613 | A/C | *SpGA2022_003090*: *SpERF071* | Ethylene-responsive transcription factor | Exonic region | 3.77·10^-6^ |
| Shikimic acid,  iPR,  tZR | 12 | 660635 | C/G | - | Intergenic region > 60 kbp away from next coding sequence | - | 7.97·10^-12^,  1.22·10^-14^,  1.36·10^-13^ |
| Shikimic acid | 14 | 1136868 | A/C | *SpGA2022_0055209*: *SpPEX19-1* | Peroxisome biogenesis protein 19-1 | Exonic region | 3.45·10^-6^ |
| Shikimic acid,  iP | 17 | 3839011 | A/T | *SpGA2022_019301*: *SpPID* | Protein kinase PINOID | 4235 bp downstream | 2.14·10^-6^,  1.32·10^-6^ |
| Chlorogenic acid | 3 | 3707844 | A/G | *SpGA2022_005797* | Unknown function | Exonic region | 7.92·10^-7^ |
| Chlorogenic acid | 4 | 3248185 | A/G | *SpGA2022_007066*: *SpNOD19* | Nodulin19 | Intronic region | 7.69·10^-10^ |
| Chlorogenic acid | 7 | 2101352 | G/C | *SpGA2022_010583* | Unknown function | Intronic region | 3.51·10^-6^ |
| Chlorogenic acid | 9 | 2006394 | C/G | *SpGA2022_053849*: *SpCYP71AU50* | Cytochrome P450 71 | 7584 bp upstream | 1.34·10^-8^ |
| Chlorogenic acid | 11 | 3813073 | G/A | *SpGA2022_015013*: Homolog of *At1g56130* | Probable LRR receptor-like serine/threonine-protein kinase | Exonic region | 3.30·10^-6^ |
| Coumaric acid | 17 | 2596990 | T/C | *SpGA2022_055775*: *SpHAK12* | Putative potassium transporter 1 | 3400 bp upstream | 6.71·10^-7^ |
| Sinapic acid, Luteolin | 1 | 9102932 | G/A | *SpGA2022_050859*: *SpTMEM184A* | Transmembrane protein 184A | 5772 bp downstream | 3.43·10^-6^,  8.65·10^-7^ |
| Sinapic acid | 3 | 2333724 | C/G | - | Intergenic region > 60 kbp away from next coding sequence | - | 3.88·10^-6^ |
| Apigenin | 2 | 5607569 | C/T | *SpGA2022_051344* | Unknown function | 125 bp upstream | 2.78·10^-6^ |
| Apigenin | 2 | 7491771 | C/T | *SpGA2022_005323* | Unknown function | 342 bp upstream | 4.31·10^-7^ |
| Cyanidin-3-O-glycoside | 6 | 1885388 | A/G | *-* | Intergenic region > 40 kbp away from next coding sequence | - | 2.85·10^-7^ |
| iP | 13 | 1671306 | C/T | *SpGA2022_054960*: *SpPNM1* | Pentratricopeptide repeat-containing protein PNM1 | 10531 bp upstream | 2.63·10^-9^ |
| iP | 6 | 2254891 | T/C | - | Intergenic region > 20 kbp away from next coding sequence | - | 6.50·10^-9^ |
| iP | 18 | 4022067 | A/C | *SpGA2022_019833*: homolog of *AT1G59675* | F-box containing protein | Exonic region | 4.34·10^-6^ |
| iP | 9 | 6998734 | A/G | *SpGA2022_013565*: *SpBACOVA_02659* | Beta-glucosidase BoGH3B | Exonic region | 9.33·10^-7^ |
| iP | 2 | 8321040 | G/A | *SpGA2022_051428*: *SpTOM1* | Tobamovirus multiplication protein 1 | Intronic region | 8.15·10^-7^ |
| iP | 4 | 4459285 | G/A | *SpGA2022_007215*: *SpSPPL2* | Signal peptide peptidase-like 2 | Intronic region | 1.04·10^-6^ |
| iP | 4 | 7453011 | T/C | *SpGA2022_007797* | Unknown function | 1567 bp upstream | 1.45·10^-6^ |
| iP,  iPR,  tZ,  tZR | 5 | 4676913 | C/T | *SpGA2022_008667*: *SpHPL* | Fatty acid hydroperoxide lyase | 13696 bp upstream | 2.29·10^-9^, 2.65·10^-9^, 7.34·10^-11^, 1.14·10^-7^ |
| iP,  iPR,  tZ | 4 | 6589981 | A/C | *SpGA2022_052203*: *SpFRO6* | Ferric reduction oxidase 6 | 3182 bp upstream | 1.02·10^-13^,  4.75·10^-6^,  7.01·10^-8^ |
| iP,  iPR,  tZ,  tZR | 7 | 4346941 | G/A | *SpGA2022_010966* | Unknown function | Intronic region | 2.49·10^-13^,  1.45·10^-8^,  2.90·10^-11^,  8.57·10^-^**^9^** |
| tZ | 6 | 2254891 | T/C | - | Intergenic region > 20 kbp away from next coding sequence | - | 2.46·10^-6^ |
| tZ | 17 | 2596990 | T/C | *SpGA2022_055775*: *SpHAK12* | Putative potassium transporter 1 | 3400 bp upstream | 5.33·10^-7^ |
| cZR | 9 | 2886292 | T/C | *SpGA2022_053891*: *SpPCMP-H12* | Pentratricopeptide repeat-containing protein | 6532 bp downstream | 3.87·10^-8^ |
| IAA | 1 | 4153168 | G/A | *SpGA2022_002976*: *SpMYBC1* | Transcription factor MYBC1 | Exonic region | 4.96·10^-7^ |
| IAA | 1 | 7513300 | G/A | *SpGA2022_050798*: *SpDPE2* | 4-alpha-glucanotransferase DPE2 | Exonic region | 2.44 ·10^-6^ |

Abbreviations: ABA – Abscisic acid, cZ – cis-Zeatin, cZR – cis-Zeatin riboside, IAA – Indole-3-acetic acid, iP – Isopentenyladenine, iPR – Isopentenyladenine riboside, JA – Jasmonic acid, JA-Ile – Jasmonic acid-isoleucine conjugate, SA – Salicylic acid, tZ – trans-Zeatin, tZR – trans-Zeatin riboside
